# Ibrutinib disrupts blood-tumor barrier integrity and prolongs survival in rodent glioma model

**DOI:** 10.1186/s40478-024-01763-6

**Published:** 2024-04-08

**Authors:** Sanghee Lim, Minhye Kwak, Jeonghan Kang, Melissa Cesaire, Kayen Tang, Robert W. Robey, William J. E. Frye, Baktiar Karim, Donna Butcher, Martin J. Lizak, Mahalia Dalmage, Brandon Foster, Nicholas Nuechterlein, Charles Eberhart, Patrick J. Cimino, Michael M. Gottesman, Sadhana Jackson

**Affiliations:** 1 Develomental Therapeutics and Pharmacology Unit, Surgical Neurology Branch, National Institute of Neurologic Disorders and Stroke (NINDS), NIH, Building 10, Room 7D45, 10 Center Drive, Bethesda, MD 20892 USA; 2 Laboratory of Cell Biology, Center for Cancer Research, National Cancer Institute (NCI), NIH, Bethesda, MD 20892 USA; 3 Molecular Histopathology Laboratory, Frederick National Laboratory, Leidos Biomedical Research, Frederick, MD 21702 USA; 4 NIH MRI Research Facility and Mouse Imaging Facility, National Institute of Neurologic Disorders and Stroke (NINDS), NIH, Bethesda, MD 20814 USA; 5 Department of Pathology, Johns Hopkins University School of Medicine, Baltimore, MD USA; 6 Neuropathology Unit, Surgical Neurology Branch, National Institute of Neurologic Disorders and Stroke (NINDS), NIH, Bethesda, MD 20892 USA

**Keywords:** Glioma, Blood-tumor barrier, Endothelial cells, Ibrutinib, Doxil

## Abstract

In malignant glioma, cytotoxic drugs are often inhibited from accessing the tumor site due to the blood-tumor barrier (BTB). Ibrutinib, FDA-approved lymphoma agent, inhibits Bruton tyrosine kinase (BTK) and has previously been shown to independently impair aortic endothelial adhesion and increase rodent glioma model survival in combination with cytotoxic therapy. Yet additional research is required to understand ibrutinib’s effect on BTB function. In this study, we detail baseline BTK expression in glioma cells and its surrounding vasculature, then measure endothelial junctional expression/function changes with varied ibrutinib doses in vitro. Rat glioma cells and rodent glioma models were treated with ibrutinib alone (1–10 µM and 25 mg/kg) and in combination with doxil (10–100 µM and 3 mg/kg) to assess additive effects on viability, drug concentrations, tumor volume, endothelial junctional expression and survival. We found that ibrutinib, in a dose-dependent manner, decreased brain endothelial cell–cell adhesion over 24 h, without affecting endothelial cell viability (*p* < 0.005). Expression of tight junction gene and protein expression was decreased maximally 4 h after administration, along with inhibition of efflux transporter, ABCB1, activity. We demonstrated an additive effect of ibrutinib with doxil on rat glioma cells, as seen by a significant reduction in cell viability (*p* < 0.001) and increased CNS doxil concentration in the brain (56 ng/mL doxil alone vs. 74.6 ng/mL combination, *p* < 0.05). Finally, Ibrutinib, combined with doxil, prolonged median survival in rodent glioma models (27 vs. 16 days, *p* < 0.0001) with brain imaging showing a − 53% versus − 75% volume change with doxil alone versus combination therapy (*p* < 0.05). These findings indicate ibrutinib’s ability to increase brain endothelial permeability via junctional disruption and efflux inhibition, to increase BTB drug entry and prolong rodent glioma model survival. Our results motivate the need to identify other BTB modifiers, all with the intent of improving survival and reducing systemic toxicities.

**Supplementary Information:**

The online version contains supplementary material available at 10.1186/s40478-024-01763-6.

## Introduction

One of the crucial treatment challenges of glioblastoma is the restrictiveness of the blood–brain barrier/blood-tumor barrier (BBB/BTB). Glioma progression has been linked to BTB (blood- disruption, causing increased permeability due to structural changes from angiogenesis, astrocytic end feet displacement, and neuronal death [1]. Specifically in high-grade gliomas, structural abnormalities across the BTB can lead to extravasation of blood contents, including solutes, antibodies, fluorescent markers, and immune cells [2]. However, the BTB demonstrates heterogeneous permeability. Specifically, high-grade gliomas have certain intact sections of BTB, which aid in continued tumor growth, invasion, and limitation of therapeutic drug penetration, leading to disease progression and treatment resistance [3]. To overcome these challenges, it is essential to identify BTB modulators that can transiently increase permeability and help the entry of chemotherapy or directed cytotoxic agents in such restricted areas.

The BBB is one of the main components of the neurovascular unit which includes endothelial cells, pericytes, and astrocytic end feet working harmoniously. In a healthy person, the BBB maintains the central nervous system (CNS) homeostasis by regulating the entry and exit of drugs, molecules, and toxins [4]. The key player of the BBB is endothelial cells, mesodermally derived, modified simple squamous epithelial cells that comprise blood vessel walls and are in the most direct contact with blood that circulates throughout the brain [5]. Mechanisms of BBB permeability are mostly centered around the expression and function of endothelial tight junctions along with membrane transport proteins, such as ATP binding cassette (ABC) transporters. These endothelial cells are held together by bicellular and tricellular junction proteins that aid in communication for paracellular transport of drugs, cells and immunologics. Bicellular junctions include the claudin proteins (claudin-1, claudin-3, claudin-5), zonula occluden (ZO-1, ZO-2) and tricellular junctions include (angulin-1/LSR, tricellulin/MarvelD2). Brain endothelial cells also express exceptionally high levels of nutrient transport proteins such as ABC transporters, mainly localized on the luminal side of the vasculature, to assist with pumping specific cytotoxic chemotherapies out of the cell [6, 7]. Both endothelial cells and malignant glioma cells have been reported to express high levels of ABC transporters, specifically P-glycoprotein (P-gp) (encoded by the—ABCB1 gene), ABCG2 and ABCC4 [8, 9]. Overall, these mechanisms are regulated through various modalities, including direct communication with the CNS and neuroimmune modulators released by nearby cells. The dynamic nature of the BBB allows for permeability adjustment in response to changes in the microenvironment, especially in the presence of malignant glioma cells [10, 11].

Ibrutinib is an FDA-approved B-cell lymphoma/lymphocytic leukemia agent that inhibits BTK (Bruton tyrosine kinase) activation, leading to decreased B-cell receptor signaling and decreased proliferative potential. In cardiac endothelium, ibrutinib inhibits vascular cell adhesion, platelet aggregation, and the associated inflammatory responses that occur during endothelial cell activation [12]. Additionally, ibrutinib has properties that favor BBB permeability, with a low molecular weight (440 Da), nonpolar characteristics, and hydrophobicity [12, 13]. Previous studies demonstrated its ability to inhibit downstream cell survival pathways, synergize with radiotherapy, impair glioma-derived pericyte coverage, and prolong survival in mouse glioma models when combined with etoposide [14–16]. However, little is understood about its effect on brain endothelial junctional tightness, its interaction with brain capillary endothelial cell ABC transporters, or influence on CNS pharmacokinetics [17].

In this study, we demonstrated the effect of ibrutinib on brain endothelial junctional expression and integrity, while also detailing its ability to decrease glioma progression in combination with a chemotherapy agent with limited CNS entry. These studies specifically focused on the BTB’s supportive role can serve as a window into understanding the steps required for malignant glioma progression and disease severity, with the overall intent to improve treatment options.

## Materials and methods

### Cell culture and reagents

Rat brain microvascular endothelial cells (RBMVEC) were cultured using manufacturer instructions (Cell Application, CA). Cells were grown for 5–7 days for all experiments to establish a monolayer. Rat high grade glioma cells (S635) were obtained as a gift from D. Bigner Lab (Brain Tumor Center, Duke University) [18]. They were maintained in Dulbecco’s Modified Eagle Medium (DMEM) (Corning,MA) supplemented with 10% fetal bovine serum (FBS) (Gibco, NY) and 1% Penicillin Streptomycin (10,000 U/mL) (Gibco, NY). MDR-19 cells that overexpress human P-gp were used for ABCB1 flow cytometry functional studies with rhodamine with and without valspodar, as has been described previously [19]. Ibrutinib (Medchem express, NJ) was dissolved in DMSO*.* Injectable Doxil (Baxter Healthcare, IL) was used for in vitro and in vivo treatments.

### Impedance and migration assay

To measure cell–cell integrity and migration, an Agilent xCELLigence Real-Time Cell Analysis (RTCA) DP (dual purpose) instrument was used to quantify cell–cell impedance or cell migration according to manufacturer protocol. Specifically, cells were seeded in a 16-well xCELLigence E plate (Agilent, CA) or (CIM) plates (Agilent, CA) at a concentration of 60,000 cells/well. Cells were cultured overnight, followed by 3 h of serum starvation, and treated with ibrutinib (0, 1, 5, 10 µM). Cell index, via gold microelectrode biosensors, was measured as an indicator of impedance or migration from 0 to 48 h maximum, as described previously [20]. Specifically, for migration studies, cells were plated in the upper CIM-plate chamber. The bottom surface of the upper chamber was composed of a microporous membrane that allows cell migration. Gold electrodes on the underside of the membrane detect adherent cells. The impedance microelectric sensor on the porous membrane automatically detected cells as they migrated through the porous membrane and attached to the impedance microelectrode in the lower chambers. Changes in the unitless parameter, cell index, were measured in real-time by xCELLigence software.

### Quantitative polymerase chain reaction analysis

Total RNA was isolated from RBMVECs cell lines using RNeasy Mini isolation kits (Qiagen), and 0.5 μg of RNA was reverse transcribed into complementary cDNA using Superscript III First-strand SuperMix (Thermo Fisher Scientific). cDNA (2 μL) was used for qPCR using SYBR Green PCR mast mix (Applied Biosciences) and primers (Additional file 3: Table 1) with a Vii A 7 Real-Time PCR system (Applied Biosciences). Gene expression levels were determined by the 2^−ΔΔCt^ method. All experiments were performed in triplicate and normalized to 18 s rRNA.

### Small Interfering RNA Knockdown of BTK

RBMVECs were incubated with small interfering (si)RNAs (50 nmol/L) to BTK or nontargeting siRNAs (Thermo Fisher Scientific). Briefly, 0.5 × 10^6^ cells were transfected with Lipofectamine 2000 (Invitrogen) and placed in a complete medium for 24 h per manufacturer protocol. BTK2 exhibited the highest knockdown efficiency after 24 h, evaluated by qPCR, and was selected for downstream studies as described below.

### Cell viability assay

Cells were seeded in a 96-well plate at 0.5–4 × 10^4^ cells/well for 24 h. Ibrutinib (0, 1, 5, 10 µM) or doxil (10 uM and 100uM) as in figure was treated for 24 h, and viability was measured using the CellTiter-Glo assay per manufacturer’s protocol (Promega, WI), and luminescence was recorded by a luminometer (BioTek, VT).

### Immunoblotting

RBMVECs were plated at 1 × 10^6^ cells per well in a 6-well plate and grown until confluent. Cells were treated with 10 µM of ibrutinib for different hours. Cells were lysed with a mixture of RIPA buffer (Thermo Fisher Scientific, MA), protease inhibitor cocktail (Thermo Fisher Scientific, CA), sodium orthovanadate (BioLab, CA), and PMSF (Cell signaling, CA). Protein concentration was measured using the Bradford method (Bio-rad, CA). For each sample, 20 µg of denatured protein was loaded on 10% NuPage Bis–Tris gels (Thermo Fisher), a PVDF membrane was used, and proteins were transferred. The membrane was incubated in blocking buffer (Licor, NE) for 1 h at room temperature and incubated with primary antibody (Additional file 4: Table 2) overnight at 4 °C. Images were detected by Odyssey Imager (Licor).

### Immunofluorescence and electron microscopy

RBMVECs were seeded on 8 well chamber slides (Corning, MA), coated with cell attachment factor, and cultured until confluent. Cells were treated with 10 µM of ibrutinib for 4 h or 24 h. Cells were fixed with 4% formaldehyde for 20 min, followed by phosphate buffered solution (PBS) washed 3 times, and then permeabilized with 0.5% Triton X-100 for 5 min at room temperature. Cells were blocked with 1% bovine serum albumin (BSA) for 1 h at room temperature and incubated at 4 °C overnight with primary antibodies anti-ZO-1 (Thermo Fisher Scientific, CA). Slides were washed with phosphate-buffered saline, incubated with Alexa Fluor secondary antibodies (Life Technologies, CA) and, mounted using Prolong-Gold Antifade with DAPI, and analyzed with Leica Stellaris microscope.

Rat brain endothelial cells were fixed with 4% glutaraldehyde in 0.1 M cacodylate buffer at pH 7.4 for 30 min to 1 h at room temperature and then stored at 4 °C. Samples were post-fixed with 1% osmium tetroxide in 0.1 M cacodylate buffer for 1 h on ice and stained with 1% uranyl acetate in 0.1 N acetate buffer at pH 5.0 overnight at 4 °C. Samples were then dehydrated in a graded series of ethanol and embedded in epoxy resins. Thin sections were cut at approximately 70 nm and counterstained with lead citrate. Images were photographed with a bottom mounted digital CCD camera (AMT XR-100, Danvers, MA, USA) on a JEOL 1200 EX transmission electron microscope at 60 kV. At least 6 varied regions of the section were evaluated to determine presence of differences with and without treatment.

### Caspase 3/7 apoptosis assay

Apoptosis was assessed with the Caspase-Glo 3/7 Assay (Promega). S635 cells were grown in 96-well plates and treated with ibrutinib alone (10 µM), doxil alone (10 µM, 100 µM) and ibrutinib (10 µM) with doxil (10 µM, 100 µM) added for 24 h [21]. The following day, 100 μL of Caspase-Glo 3/7 was added and incubated for 2 h at 37 °C. Luminescence was measured using a luminometer (BioTek, VT) per manufacturer specifications.

### In vitro permeability assay/Rhodamine 123 uptake assay

For the permeability assay, 3 × 10^5^ RBMVEC cells were plated in the upper chamber of 12-well tissue culture inserts (Corning, ME) pre-coated with gelatin (Fisher, NH) for 5 days. Approximately, 10 mg/mL of sodium fluorescein (MW 376) was added to endothelial cells then treated with PBS or 10 µM ibrutinib for 24 h. Low chamber of sodium fluorescein measured after 24 h was evaluated using a luminometer (BioTek, VT) per manufacturer specifications. Fluorescent rhodamine intracellular accumulation was measured by FACS Canto II flow cytometer (BD Biosciences, CA). RBMVEC and S635 cells were trypsinized and incubated for 30 min with a medium containing 0.5 µg/mL rhodamine 123 (in phenol red-free DMEM) in the absence or presence of the known P-gp/ABCB1 inhibitor valspodar (3 µg/mL) or varying concentrations of ibrutinib (1, 5, 10 µM). The medium was then removed and replaced with a rhodamine-free medium, continuing with or without inhibitors for an additional 1 h. Subsequently, intracellular fluorescence was analyzed with FlowJo software (v 10.6.1, Tree Star, Inc, Ashland, OR).

### Rat glioma-allograft model and drug treatment

All animal procedures followed the National Institutes of Health Animal Use and Care (ACUC) Guidelines. Female Fischer 344 rats (5 weeks old, ENVIGO) were anesthetized by isoflurane inhalation (induced with 5% and maintained with 2% isoflurane in oxygen) and positioned in a stereotactic head frame. A total of 1 × 10^4^ S635 glioblastoma cells (gifted from Duke University, Durham, NC) in 3 μl of PBS were implanted into the right hemisphere (2 mm anterior and 2 mm lateral to the bregma; 4 mm depth) using 33-gauge Hamilton syringe connected to UMP3T pump (WPI, Sarasota, FL) at a flow rate of 0.5 μl/min. Seven days after implantation, rats were randomized into 4 groups (4–7 animals per group) and subjected to 25 mg/kg of ibrutinib gavage injection on days 7–14 and 3 mg/kg of doxil tail vail injection on days 7 and 14.

### Magnetic resonance imaging and analysis

Magnetic resonance imaging (MRI) was performed in a 4.7-Tesla Bruker Avance III MRI system with ParaVision 6 software (Bruker, Billerica, MA). After anesthesia, the rats were placed on a custom cradle equipped with a nose cone and bite bar to reduce ancillary head motion. Before the MRI, a tail vein line was placed and kept open with heparinized saline. MRI scans used 0.1 mmol/kg Gadovist™ as a T1-weighted contrast agent. Following an initial set of localizer scans, a 1 mm axial slices were scanned that covered the brain. The MRI data was analyzed using the ImageJ software (version 1.53, NIH).

### Quantification of Doxil distribution

Two hours after the final doxil administration on day 14, intracardiac blood was collected under deep anesthesia, and animals were perfused with saline to rinse unabsorbed doxil. The tumor-bearing brain, and contralateral counterparts were harvested, homogenized, and incubated in acidified ethanol for 1 day at 4 °C to extract doxil. Samples were centrifuged at 16,000 g for 25 min at 4 °C and supernatant was extracted. Doxil concentration in the brain and plasma were quantified using a fluorometric assay with a microplate reader (Cytation 5, BioTek, VT) as previously described [22]. Fluorescence of free doxorubicin release from doxil measurements were quantified approximately 2 h after doxil administration, as previous studies have outlined optimized timing [23, 24].

### Histology

To evaluate the histological effects of each treatment, four of the animals per group were sacrificed on 14 days after tumor implantation. The animals were deeply anesthetized and brains were fixed via transcardial perfusion with 4% paraformaldehyde (PFA). The brain was then collected, dehydrated by immersion in 30% sucrose solution, embedded in OCT compound, and sectioned into 10 μm using a cryostat (Leica Biosystems, Buffalo Grove, IL). The coronal sections were mounted on glass slides and were stained using hematoxylin and eosin staining kit (Abcam) as manufacturer’s instruction. Whole slide images were obtained using fluorescent stereo microscope (M165FC, Leica Biosystems).

### Dextran permeability and Immunohistochemistry

Fourteen days after tumor implantation, 6 mg/kg of Texas Red-conjugated 3 kDa dextran (TRD, ThermoFisher) was injected intravenously through the tail vein. TRD was allowed to circulate for 20 min, animals were exsanguinated by intracardiac perfusion of PBS with subsequent perfusion of 4% PFA solution for brain tissue fixation. Extracted brains were embedded in paraffin and sectioned at 5 μm thickness. Formalin-fixed sections were then subjected to either TRD staining or immunohistochemical (IHC) staining. Whole slide images were obtained at high resolution, 20×*g* using an AT2 scanner (Aperio, Leica Biosystems, Buffalo Grove, IL). Double staining for CD31/Claudin5 were performed on the Leica Biosystems Bond RX autostainer using the Bond Polymer Refine Kit (Leica Biosystems DS9800), with omission of the Post Primary reagent, DAB and Hematoxylin. Slides were double stained for CD31 (Abcam ab28364, 1:100), and Claudin-5 (Invitrogen 35-2500, 1:50). Images were captured using the Aperio Scanscope FL whole slide scanner (Leica Biosystems) into whole slide digital images and OPAL Fluorophore 520 (AKOYA) as per previously published [25]. Image analysis was performed using Halo imaging analysis software (v3.3.2541.423, Indica Lab’s, Corrales, NM) using Halo algorithm (Area Quantification FL v2.3.4) which were used to quantify positive area of TRD, CD31, and Claudin5.

### Tissue microarrays (TMA)

The use of human tissues for these studies was approved by Johns Hopkins University School of Medicine Institutional Review Board and National Institutes of Health Material Transfer Agreement and adhered to the policies and practices of the Declaration of Helsinki. One TMA containing 80 adult and pediatric patients with a diagnosis of glioblastoma from surgical and autopsy cases in the years 1981–2013 at Johns Hopkins Hospital and generated by the Johns Hopkins TMA facility, as previously published [26, 27]. Internal control tissue of lymph node, colon, thyroid, tonsil, muscle, brain, pancreas, placenta, liver, intestine, kidney, stomach, bladder, skin, vasculature, appendix, endocervical, breast, and prostate were included. The TMAs were stained for BTK to detect expression in tumor and/or endothelium. Deparaffinization, antigen retrieval and immunostaining were performed with a Leica Bond Max Section from each block and were stained with H&E or BTK (1:50, Sigma Aldrich, HPA001198). Imaging was performed with a Leica Aperio Versa 200 slide scanner. Image quantification scoring was performed blinded by a single neuropathologist (PJC) with a sampling of four fields per patient sample, and 10–15 cells analyzed per field. BTK was quantified within the nuclei of cells, and total cell area quantified for staining with scoring of 0–3 intensity comparing tumor versus tumor vasculature expression to known positive high BTK expressing lymph tissue.

### Whole genome sequencing analysis

Whole genome extraction performed on S635 glioma cells using the Promega kit as instructed (655000L066123) and then processed by Azenta Life Sciences. The tools available in the CLC Genomic Workbench v23 (www.qiagen.com) were used under default parameters unless otherwise noted. Fastq files were imported into the Workbench using the “Illumina High-Throughput Sequencing import” tool. To inspect the quality of the sequence reads, the “QC for Sequencing Reads” tool was used. To trim remove adaptor sequence (CTGTCTCTTATACACATCT), dynamically remove low quality sequence (Quality lmimt = 0.01), and hard trim remove the first 10nt from the 5′ end and the last 1nt from the 3′end, the “Trim Reads” tool was used. For reference alignment to Rat (mRatBN7.2.111), the “Map Reads to Reference” tool was used. Post alignment, duplicate aligned reads were removed then the alignments refined using the “Remove Duplicate Mapped Reads” tool and “Refine Read Mapping” tool respectively. Insertions, deletions, and structural variants were then called using the refined alignments using the “InDels and Structural Variants” tool. Insertion and Deletions called were then passed to the “Prepare Guidance Track” tool to generate a guidance track. This guidance track was then used as input into the “Local Realignment” tool along with the refined alignments to generate local realigned and refined alignments. These local realigned and refined alignments were then used as input into the “Low Frequency Variant Detection” tool to call single nucleotide variations (snvs), multiple nucleotide variations (mnvs), insertions, and deletions. For annotating the variants called regarding functional consequences, the “Amino Acid Changes” and “Predict Splice Site Effect” tools were used. To estimate copy number, a tool not supported in the Workbench, called “Control-FREEC” (https://boevalab.inf.ethz.ch/FREEC/) was applied to the local realigned and refined alignments over a range of ploidy (ploidy = 2, 3, 4, 5, 6, 7, 8). Based on the ploidy esitmate returned from this tool (output_ploidy = 7), variants were filtered to keep those observed to have a freqeuncy ≥ 14% and indicated by functional annotation to be non-synonomous. Surviving variants were then summarized in table form by variant type stratified by chromosome. Variants prior to filtering were also summarized in table form for select genes (Atrx, Egfr, H3f3a, H3f3b, Idh1, Idh2, Tert, Tp53).

### Statistical analyses

Data are presented as mean ± standard error of the mean, unless otherwise noted. The Student *t*-test (2-tailed), and 1-way analysis of variance with Tukey’s test evaluated statistical significance with GraphPad Prism 9.3 software (GraphPad Software, Inc).

## Results

### High bruton tyrosine kinase (BTK) expression in grade IV human gliomas

**Fig. 1:**
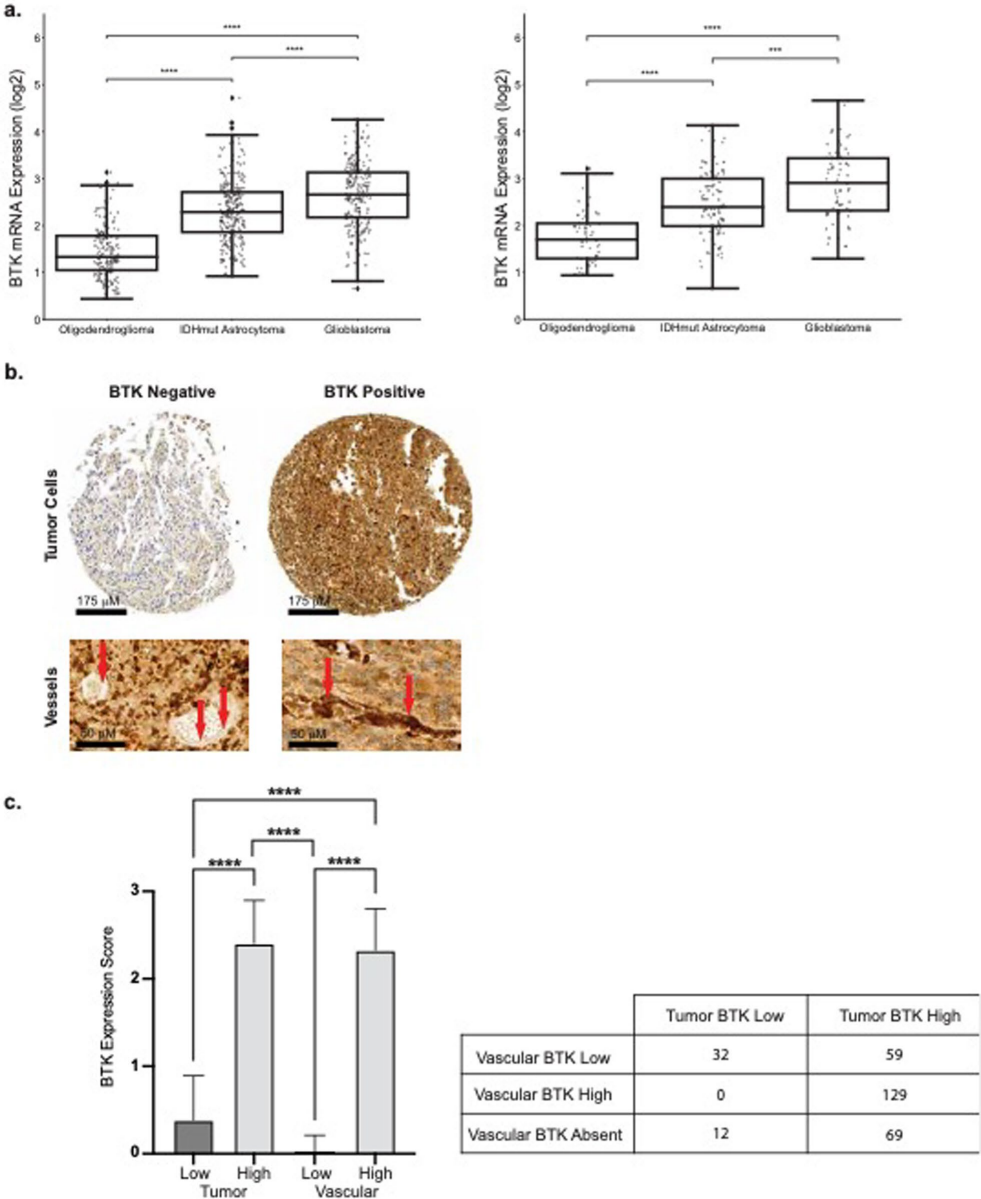
High BTK expression in glioma tumors and vasculature. High BTK expression seen in grade IV gliomas with highest expression in glioblastoma tumors as noted by CGGA (left) and TCGA (right) plotted data (**a**). Representative low and high BTK expression staining in glioblastoma TMA. Low power magnification of BTK expression seen in tumor cells and high magnification is BTK expression of endothelium. Red arrows denote vascular staining (**b**). High BTK expression scoring predominately seen in both tissue and vascular staining, with table delineating sole versus dual BTK expression, ****p < 0.001 (**c**)

Previous studies have demonstrated the high expression of BTK/BMX in glioblastoma [28, 29]. Through the use of the Chinese Glioma Genome Atlas (CGGA), and cancer genome atlas (TCGA) we demonstrated the high expression in multiple histologic diagnoses of grade IV gliomas (oligodendroglioma, IDH mutant astrocytoma and glioblastoma-GBM) in these two databases, CCGA – left, TCGA- right (Fig. 1a) [30]. These findings led us to explore BTK expression in a large TMA of both adult and pediatric glioblastoma tumor tissue (n = 80). We found that there was a predominance of high expression (≥ 2 scoring) both of the tumor cells (n = 67) and tumor vasculature (n = 49) (Fig. 1b, c) compared with known high BTK expressing lymph tissue and low expressing BTK seen in liver, colon, lung and soft tissue (Additional file 1: Fig. 1a). These findings led us to further explore the effect of BTK inhibition on both endothelium and glioma cells.

### Ibrutinib decreases endothelial cellular interactions by tight junction disruption

**Fig. 2:**
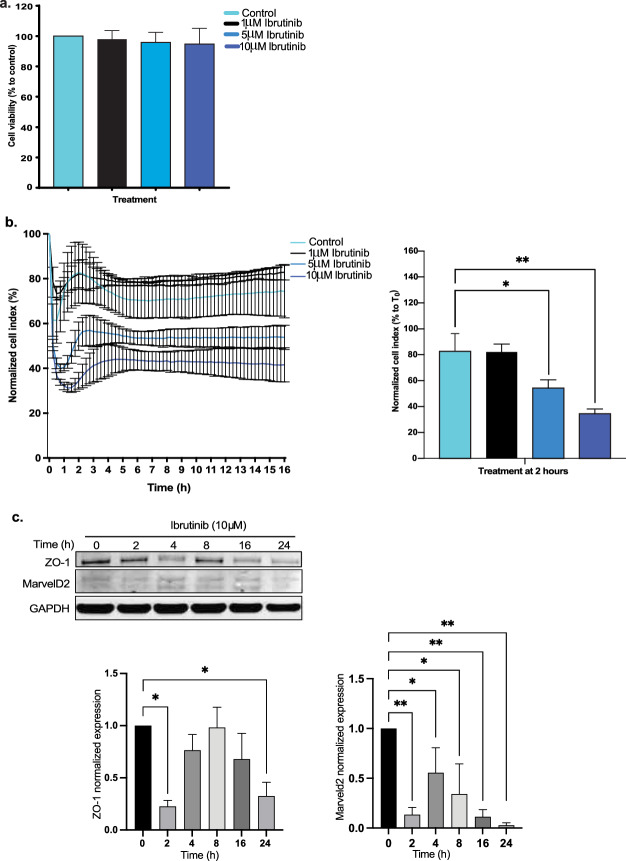

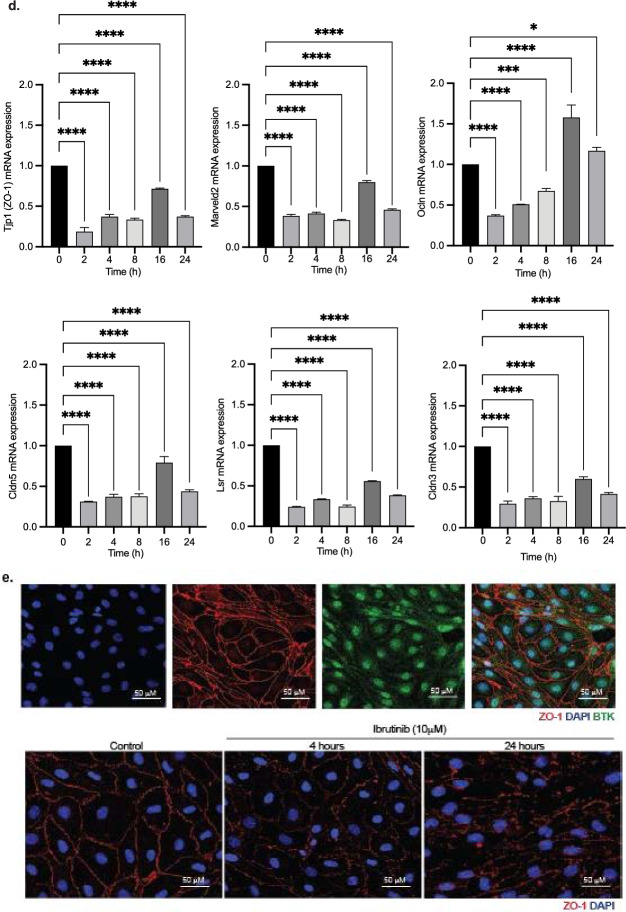

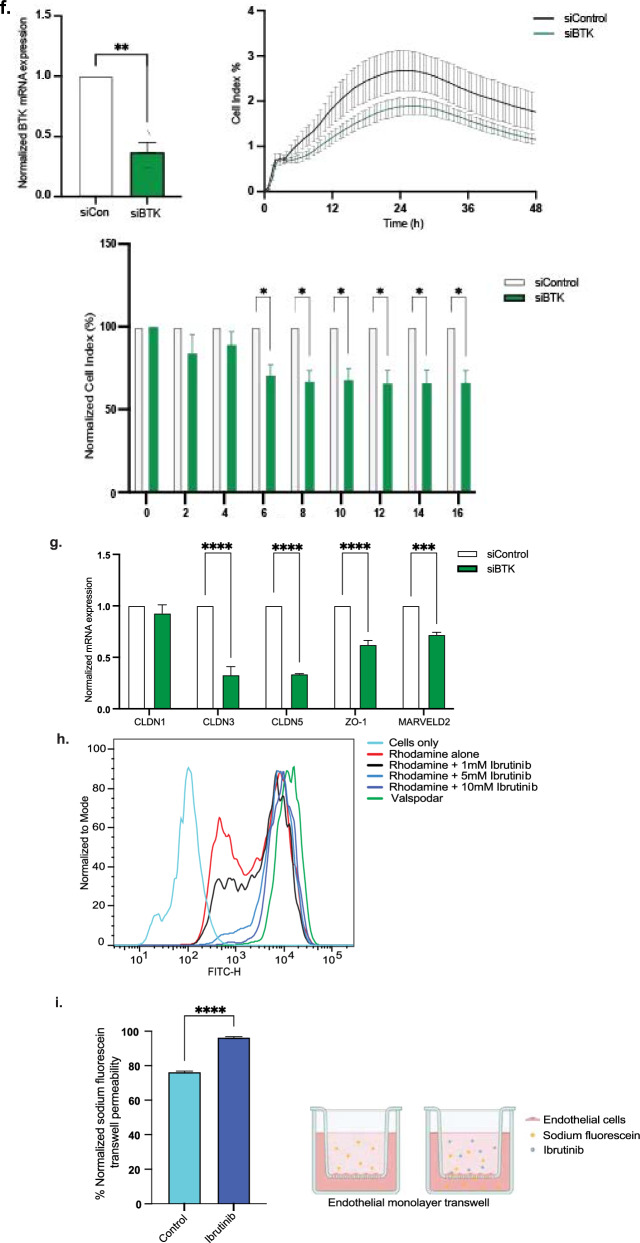
Ibrutinib disrupts brain endothelial integrity and inhibits ABC transporter function. Brain endothelial cell viability is not affected by ibrutinib treatment at varied doses (**a**). Dose-dependently, ibrutinib decreases brain endothelial cell–cell impedance, significantly 2 h after treatment seen with 5 and 10 µM with subsequent cell index plateau (*p < 0.05, **p < 0.005) (**b**). Bicellular junction protein ZO-1 and tricellular junction protein MarvelD2 was significantly decreased 2 h after 10 µM ibrutinib treatment (**c**). Junctional mRNA expression also decreased from 2 to 24 h after 10 µM treatment, without a washout period seen in tjp1 (ZO-1), MarvelD2 (tricellulin), Ocln (occludin), Cldn5 (claudin-5), Lsr (lipolysis stimulated lipoprotein receptor/angulin-1), and cldn3 (claudin-3) (**d**). High baseline BTK expression seen in both cytoplasm and nucleus of brain endothelial cells. Confirmatory immunostaining of ZO-1 expression demonstrated decreased tight junctional linear staining at 4 h, with rearrangement closer to baseline regarding adhesion expression by 24 h. (**e**). Silencing of BTK with siBTK results in decreased cell–cell impedance transiently and impaired tight junction gene expression (***p < 0.0005, ****p < 0.0001) (**f**, **g**). Ibrutinib dose-dependently inhibited Abcb1 function to increase rhodamine accumulation with higher FITC-H fluorescence measurement causing a shift of amplitude to the right, comparative to valspodar (ABCB1 inhibitor) treated cells (**h**). Monolayer endothelial cells treated with ibrutinib on transwells resulted in approximately 26% higher sodium fluorescein permeability compared with control treatment 24 h later (****p < 0.0001) (**i**)

We tested the viability of RBMVECs after 24 h of ibrutinib therapy, wich demonstrated significant reduction in cell viability over varied dosings (0, 1, 5 and 10 µM) (Fig. 2a). Using this same ibrutinib dosing, over 16 h, we measured cellular impedance of RBMVECs. Cellular adhesion/impedance measuring of endothelial cell–cell interaction was performed via gold microelectrode biosensors (covering up to 80% surface area) on the inferior aspect of a microtiter plate well (ACEA Xcelligence) and the impedance of electron flow is reported via a unitless parameter (cell index). Our brain endothelial cells reached confluence in media as measured by electrical conduction (22 mV) at the electrode-solution interface and with relative cell index of 100%. We found that upon the addition of ibrutinib, endothelial cell–cell impedance was disrupted in a dose-dependent manner (Fig. 2b). Specifically, we observed attenuated cell indices at 5 µM (1.5 fold) and 10 µM (2.4 fold) of ibrutinib 2 h after treatment, without recovery to baseline, in comparison to control and 1 µM treatment (*p < 0.05, **p < 0.005). Furthermore, we investigated the role ibrutinib plays on bicellular (occludin, claudin-3, claudin-5, ZO-1) and tricellular (tricellulin/MarvelD2, angulin-1/LSR) tight junction mRNA and protein expression (Fig. 2c, d). We found that there was a significantly reduced gene and protein expression (> 40%) approximately 2 h after ibrutinib treatment. With efforts to display baseline BTK expression, we stained our endothelial cells for BTK and ZO-1 bicellular junction protein and found high BTK expression within the cytoplasm and nucleus contrasting with ZO-1 membrane staining. Supportive findings demonstrated reduced ZO-1 expression 4 h after ibrutinib treatment compared to control, which then recovered by 24 h (Fig. 2e). Additionally, high expression of phosphorylated ERK (p-ERK) is known to play a companion role with ensuring tight junction integrity. Thus, akin to the ibrutinib effect on junctional proteins, we observed statistically significant reduced to absent p-ERK expression 0.5, 2, 4, 8 h after ibrutinib treatment, while total ERK protein expression remained unchanged on endothelium (Additional file 1: Fig. 1b) [31–33]. With use of small interfering RNA to Bruton’s tyrosine kinase (siBTK) in RBMECs, we evaluated the effect of silencing BTK (< 50%) on brain endothelial junctional integrity. We found, similar to ibrutinib therapy, significantly reduced cell–cell adhesion over time (6–16 h after plating) which can be attributed to decreased bicellular and tricellular junctional gene expression (*p* < 0.0005 and *p* < 0.0001, respectively) compared with siControl (Fig. 2f–g). To see the correlation to human brain endothelium, ibrutinib treatment also inhibited ZO-1 expression on human brain microvascular endothelial cells (HCMEC/D3) in a dose-dependent manner after 24 h (Additional file 1: Fig. 1c). For additional evaluation, we found 10 µM ibrutinib treatment also significantly decreased brain endothelial cell–cell interaction via electron microscopy, 4 h after treatment (Additional file 1: Fig. 1d).

Understanding that ABC transporter ABCB1 plays a significant role in regulating drug entry regardless of the integrity of the BBB, we next evaluated the effects of ibrutinib on efflux transport function in RBMVECs. We again observed ibrutinib dose-dependently inhibited ABCB1 function in a dose-dependent manner, with 10 µM dosing resulting in significantly higher intracellular rhodamine, ABCB1 substrate, compared to vehicle; akin to that seen with the ABCB1 inhibitor valspodar (Fig. 2h). These findings detail the ABCB1 inhibition evident from ibrutinib therapy, as a means to impair ABCB1 substrate/drug efflux activity. We also demonstrated ibrutinib’s effects are not unique only to endothelium by using ABCB1 overexpressing transfected HEK293 cells, which comparatively mimicked the inhibitory qualities of ABCB1 dose-dependently (Additional file 1: Fig. 1e);. To quantify the function of brain endothelial permeability, we measured sodium fluorescein transport (380 MW) on endothelial laden transwell plates. We observed that 10 µM ibrutinib treatment provided 26% increased permeability compared with control treatment (*p* < 0.0001); again demonstrating the endothelial disrupting effects of ibrutinib therapy (Fig. 2i).

### Ibrutinib and doxil additively reduce glioma viability

**Fig. 3:**
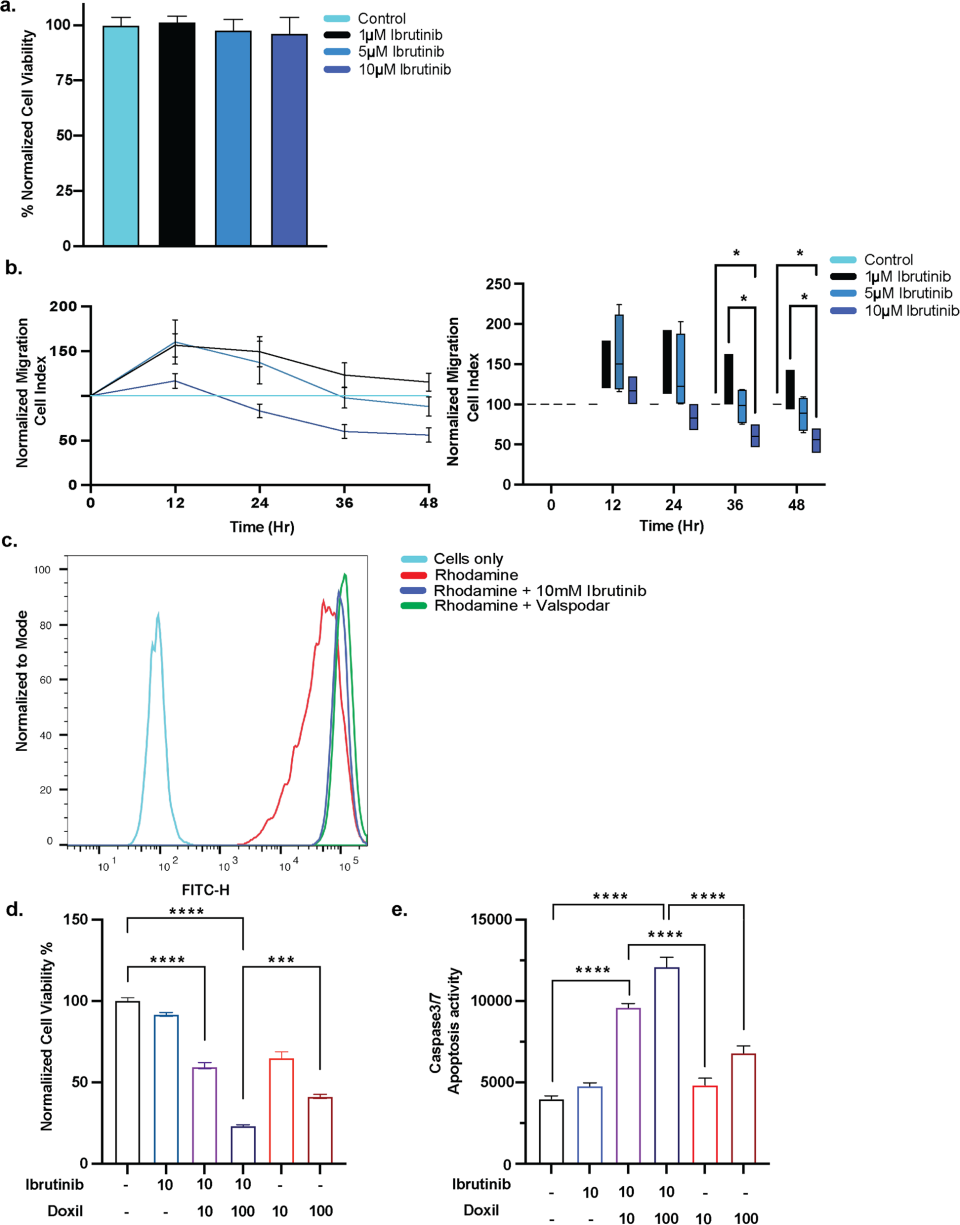
Ibrutinib hinders glioma cell migration and Abcb1 efflux and viability in combination with doxil. Varied ibrutinib dosing does not influence S635 rat glioma cell viability after 24 h (**a**). Glioma cell migration was influenced greatest by 10 µM ibrutinib 36–48 h after treatment compared with control and 1 µM therapy as evidenced by decreased cell migration to serum containing fetal bovine-serum (*p < 0.05) (**b**). Rhodamine efflux as a measure of Abcb1 function demonstrates both 10 µM ibrutinib effectively decreased efflux akin to known inhibitor valspodar (**c**). Combination therapy of ibrutinib with doxil found dose-dependent cooperation to hinder cell viability after 48 h exposure to therapy (***p < 0.001****p < 0.0001) (**d**). Combined ibrutinib (10 µM) and doxil (10 and 100 µM) resulted in increased caspase3/7 apoptosis activity compared with single therapy (****p < 0.0001) (**e**)

To evaluate the effect of ibrutinib on rat-derived glioma cells (S635), we first tested cell viability and migration. While ibrutinib showed no significant effect on glioma cell viability with varied doses, we found that time-dependently, ibrutinib significantly decreased cell migration patterns between 24 and 48 h (*p* < 0.05) as measured via cell index (Fig. 3a, b). Again, noting that ABCB1 transporter plays a crucial role in regulating drug entry to tumor cells, we investigated the effect of ibrutinib on efflux transport in glioma cells. Ibrutinib treatment resulted in impaired rhodamine dye efflux compared to vehicle treatment; with a noted shift of higher rhodamine cell accumulation, similar to that seen with ABCB1 inhibitor, valspodar (Fig. 3c).

To further identify the additive effect of ibrutinib on glioma cell viability, we combined treatment with doxil or doxorubicin. We found viability of glioma cells was reduced significantly with 10 µM ibrutinib combined with 100 µM doxil (*p* < 0.0001) 48 h after treatment compared with control. While 100 µM doxil alone decreased glioma cell viability, an additive effect was seen with combination therapy, causing approximately two times less cell viability (*p* < 0.001) (Fig. 3d) [21]. Further investigations for the cause of impaired viability, led us to conclude combined ibrutinib and doxil resulted in a statistically significant increase in caspase 3/7 apoptosis activity at varied doses (Fig. 3e).

### Ibrutinib with doxil additively improves glioma model survival

**Fig. 4:**
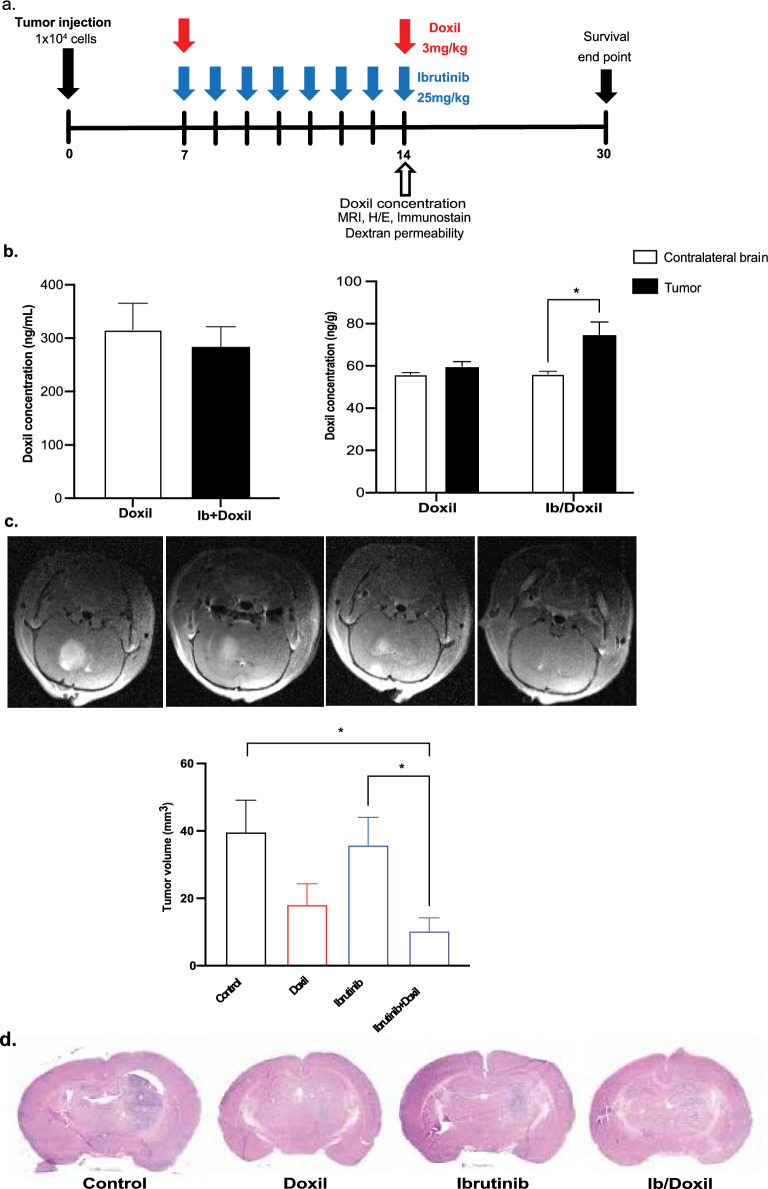

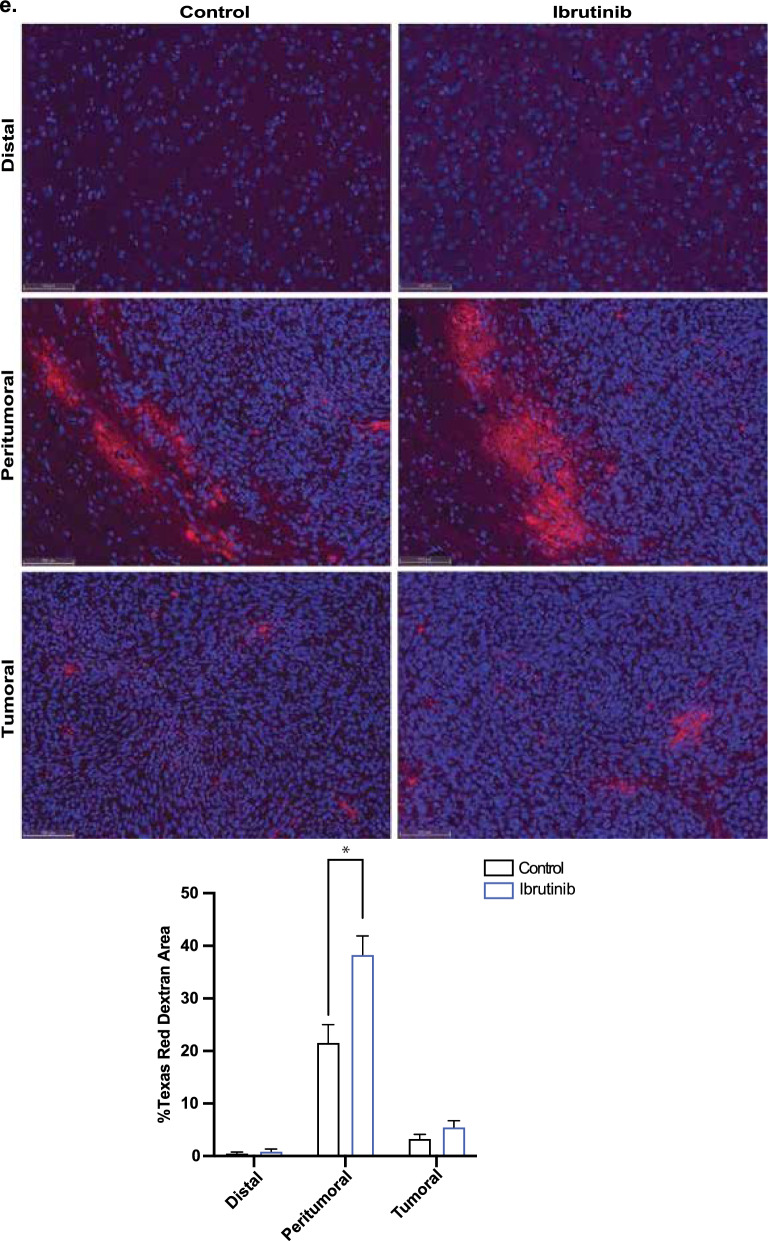

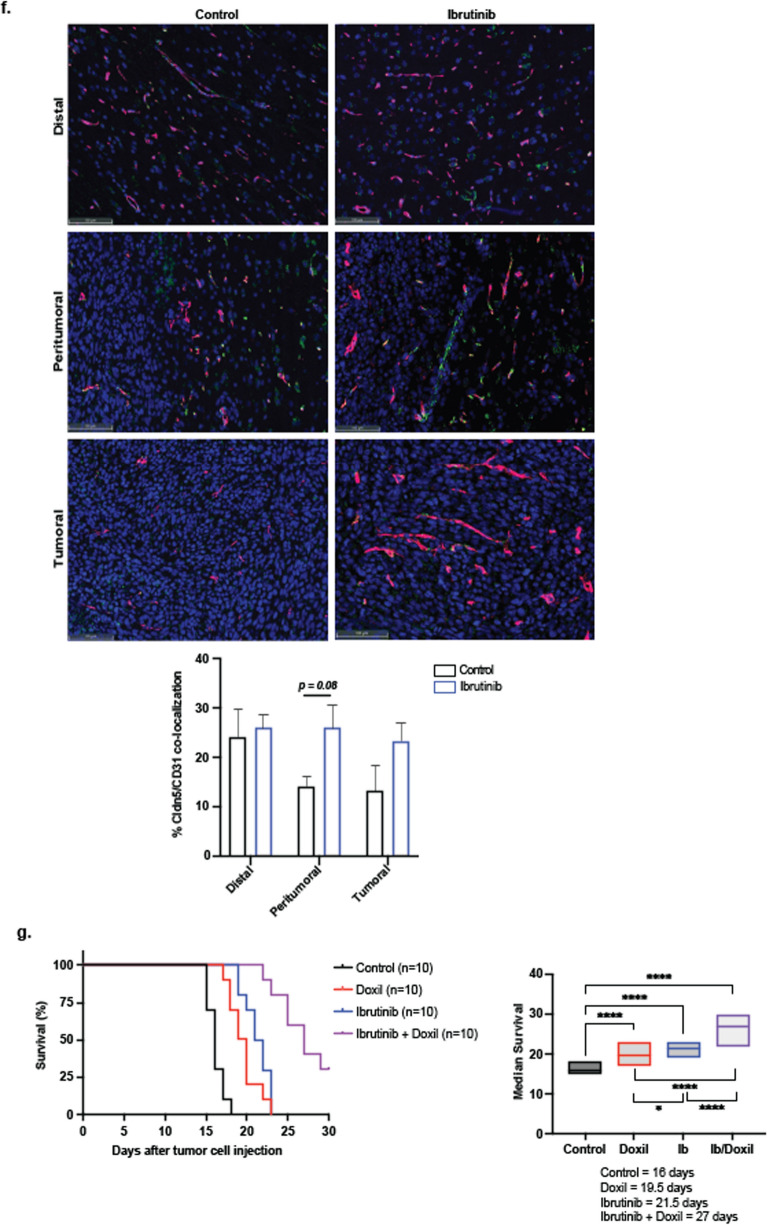
Combination ibrutinib with doxil impairs glioma model growth and prolongs survival. Treatment schema denoting repeat drug therapies, image timing, doxil blood/tissue concentrations and survival studies (**a**). Repeat ibrutinib with doxil therapy does no influence doxil plasma concentrations yet increases brain tumor doxil concentrations by approximately 32% (*p < 0.05) (**b**). Brain MRIs reflect additive ibrutinib effect of ibrutinib compared to vehicle (left panel) doxil alone (left middle panel), ibrutinib alone (right middle panel) or ibrutinib + doxil (right panel) as seen by statistically significant volumetric decrease as denoted via graphed values (*p < 0.05) (**c**). H/E staining demonstrates the lessened tumor size with doxil treatment alone and with combination therapy (**d**). 3kD dextran extravasation studies revealed ibrutinib can increase the blood-tumor barrier to larger agents, specifically within the peritumoral tissue region (*p < 0.01) (**e**). Co-staining of CD31/Claudin-5 on tumor, peritumoral and distant sites areas did not demonstrate any statistical differences in co-localized expression. (**f**). Prolonged survival seen with combination therapy with a median survival of 27 days versus 16 days for control therapy (****p < 0.0001) (*p < 0.05) (**g**)

To investigate the combined effect of ibrutinib and doxil treatment in vivo, we intracranially implanted 1 × 10^4^ S635 glioma cells in F344 5-weeks-old rats. Ibrutinib (25 mg/kg) was delivered on days 7–14, and doxil (3 mg/kg) on days 7 and 14. The half-life of ibrutinib is 4–6 h and doxil has a much longer half-life of approximately 20–48 h, which necessitated the more spaced out dosing regimen. After serial scanning, tumors were first detected on brain MRI starting day 7, while earlier imaging did not allow appreciation of sizeable tumor formation. The schematic diagram shows the timeline of treatment and experimental procedure (Fig. 4a). We first measured brain and plasma doxil concentrations 14 days after tumor implantation with without ibrutinib treatment. While there was no significant difference in plasma doxil concentrations (Fig. 4b), the concentration of the tumor injected side of brain was significantly higher (32%, *p* < 0.05) than contralateral side of the brain in the doxil treated with ibrutinib group. Brain MRI’s on day 14 agreed with these findings by showing statistically significant tumor volume declines compared with control treatment (40 ± 9 mm^3^) vs. doxil alone (19 ± 7 mm^3^) or combination therapy (10 ± 3 mm^3^) (*p* < 0.05) (Fig. 4c). These findings were in also in agreement with H/E staining which demonstrated decreased tumor size with doxil alone or combination therapy after 14 days (Fig. 4d). Ibrutinib concentration within the plasma and brain was not evaluated, as this agent was mainly used as a means to enhance CNS delivery and not provide therapeutic effects to glioma cells. Shi et al., details that 25 mg/kg dosing of ibrutinib in rodent glioma models demonstrated the best treatment effects after repeat dosing, which led us to provide the same dosing to our studies [34].

To evaluate the degree of BBB/BTB opening with ibrutinib, we perfused our rodent glioma models with 3kD dextran, with and without ibrutinib therapy at day 14 and assessed dextran extravasation within the tumor, peritumorally (0–0.2 mm from tumor bulk) and distal regions (> 0.2 mm from tumor bulk). We found that ibrutinib produced a statistically significant higher dextran accumulation within the peritumoral region compared with vehicle treatment (*p* < 0.05); denoting that ibrutinib can facilitate permeability for both small and large agents into the CNS (Fig. 4e). Yet, multiplex IHC staining for endothelium and junctional proteins with and without ibrutinib treatment showed that the co-localization of an endothelial marker, CD31, to junctional protein, Claudin-5, was not significantly different in the varied regions (Fig. 4f). Lastly, we were able to demonstrate prolonged median survival in comparison to control treatment (15 days) versus doxil alone (18 days), ibrutinib alone (21 days) or combination therapy (24 days) (*p* < 0.001), (Fig. 4g), which collectively describes the additive effect of ibrutinib with doxil to enhance BBB permeability and impair glioma growth in rodent models. Collectively, these findings demonstrate the short and long-term in vivo treatment effects of ibrutinib on brain vasculature and glioma progression, combined with cytotoxic chemotherapy.

## Discussion

Glioblastoma is known to be a fast-proliferating, aggressive brain tumor associated with a high degree of invasiveness [35, 36]. However, standard treatment, which includes surgical resection followed by chemotherapy and radiation, fails to provide extensive prolonged survival [37]. Despite several advancements in treatment, systemic and neurologic toxicities limit further radiation and chemotherapy treatments. Particularly, chemotherapy treatment has less impressive effects on GBM treatment, mainly due to the presence and function of the restrictive BBB/BTB [38, 39]. While the FDA-approved chemotherapy agent temozolomide for glioblastoma has shown some improvement in survival, with the presence of temozolomide in brain tissue only being 20% compared to plasma, again this indicates its poor CNS entry [40, 41]. Therefore, a new agent to disrupt BBB is a top priority for high grade glioma treatment.

**Fig. 5:**
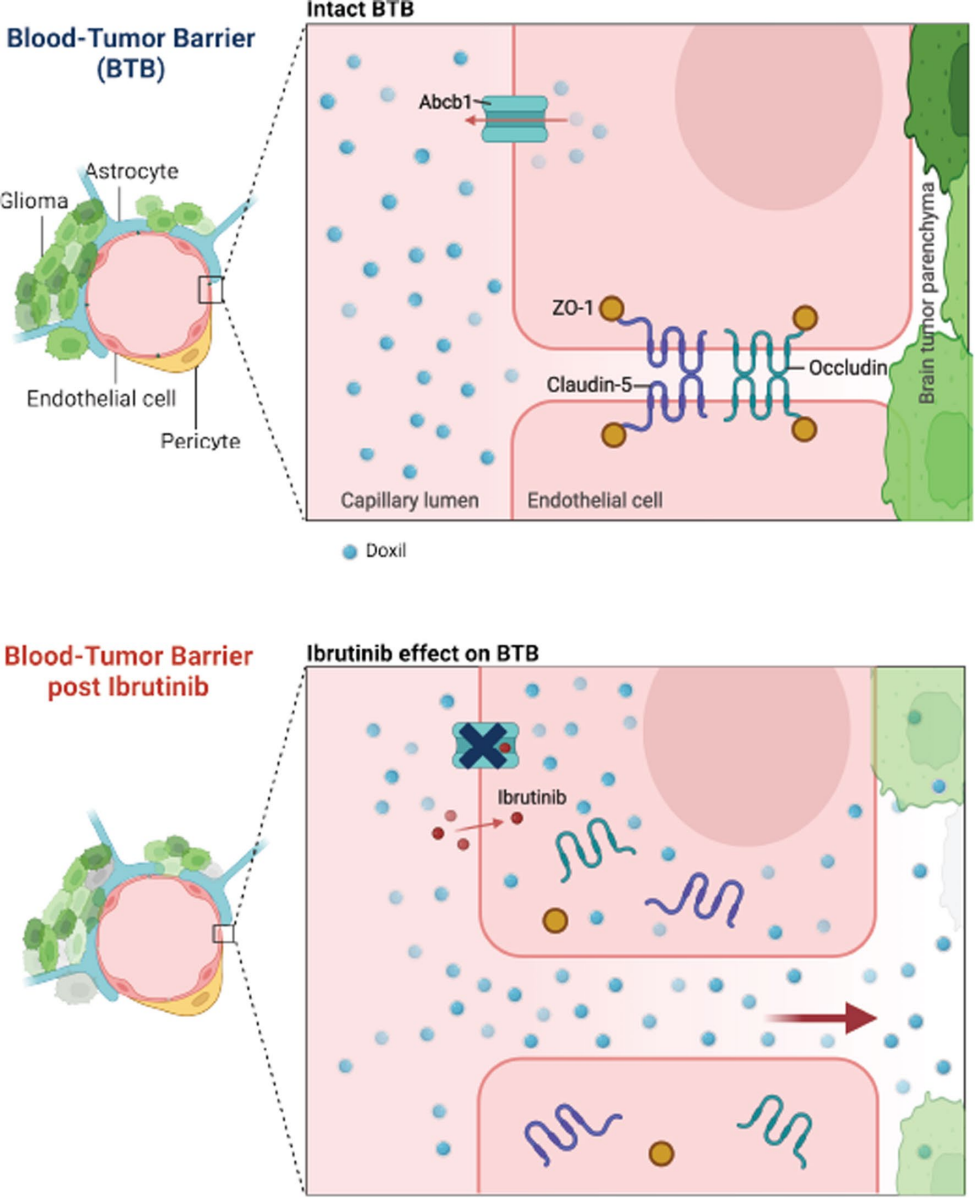
Ibrutinib aids doxil entry to hinder glioma progression. An intact blood–brain barrier limits doxil permeability through brain endothelial tight junction integrity and ABCB1 efflux activity. In the context of the blood-tumor barrier treated with ibrutinib, junctional expression is decreased, ABCB1 function is inhibited and doxil therapy entry is enhanced to delay glioma migration and growth for prolonged survival. Images created via Biorender

In this study, we were able to demonstrate the efficacy of an FDA approved lymphoma/lymphocytic leukemia agent to not only transiently disrupt brain endothelial tight junctions and inhibit efflux transport but also impair glioma migration and additive with doxil prolong rodent glioma model survival (Fig. 5). The rat derived anaplastic astrocytoma glioma line, S635, has been previously characterized histologically with high mitotic index, intratumoral necrosis, high GFAP expression and infiltrative margins with both nuclear and cytoplasmic pleomorphism [18, 42, 43]. These cells are IDH WT with non-synonymous single nucleotide variants (Additional file 5: Table 3) [44]. Collectively, these findings are influential in the field, as they demonstrate how this agent and others like it have the potential to usher in glioma cytotoxic agents that currently have limited CNS entry and influence.

Ibrutinib is both a BTK and BMX inhibitor and BMX has been specifically implicated in cell migration, tube formation and barrier function in epithelial dermal, cardiac, prostate and lung cells [45–49]. Our primary focus was to identify the effect of ibrutinib on brain endothelial cell integrity and permeability while hoping to hinder glioma proliferation, as seen in previous studies [28, 29]. While our brain endothelial cell viability studies exhibit no effects from ibrutinib, cell–cell interaction is reduced after 2 h, with notable recovery over the next 24 h. However, it was not a complete recovery, which could be due to a lack of systemic washout in this in vitro brain endothelial cell only assay. Specifically, junctional protein expression and down-stream ERK pathway proteins were found to be reduced following ibrutinib therapy, which agrees with previous studies showcasing BTK/BMX inhibition causing disruption of epithelial cell tightness [46, 47, 49]. A reduction of pERK has been shown to lead to reduced expression of tight junction proteins in the epididymis of mice, within the blood-testis barrier as reported by Kim et al*.* [50] Unfortunately, there is no one method to state whether the BBB is intact or not within these glioma models, thus evaluations of drug permeability and immunohistochemical staining of junctional markers is the best surrogate of such determinations. Combined, this data showcases ibrutinib’s ability to reduce expression of ZO-1 and other tight junctions resulting in associated attenuation of p-ERK pathway activation and ABCB1 inhibition. Previous studies have shown high BTK expression on glioma cells and ibrutinib’s efficacy to hinder lymphoma and other solid tumors progression through inhibition of the BTK/BMX pathway. Yet, no studies of ibrutinib’s effect on brain endothelium have been published, which could demonstrate a more complete picture of this drugs effects on the tumor microenvironment [12]. As such, these findings showcasing ibrutinib’s influence of brain endothelial cell–cell integrity and ABCB1 transporter function have larger implications for various neurologic, vascular and oncologic diseases. In our studies, we chose to explore the ABCB1 substrate doxil, and Zhou et al. found that ibrutinib’s use in human-derived glioma rodent models demonstrated additive cytotoxicity with etoposide (ABCB1 and ABCC1 substrate), which suggests that ibrutinib may target more than just one ABC transporter [16].

Some limitations of our studies include investigations of ibrutinib’s effects on the 1) tumor immune microenvironment, 2) long-term disease course with combination chemotherapy and 3) varied in vivo models. Our initial studies detailed the effect of ibrutinib on brain endothelium and tumor cells, as the former studies were novel and have the potential to positively benefit field knowledge related to CNS drug entry and impaired disease progression. While our studies did not explore effects of ibrutinib on the immune cells within the tumor microenvironment, previous studies have demonstrated ibrutinib’s ability to decrease glioma derived pericyte expression, which are cells known to create a formidable BBB but also demonstrate scavenger like functions when necessary [14]. In complement, Li et al., found that ibrutinib reduced CD8 + T cell exhaustion both in the in vitro setting and in BTK deficient mice, specifically by downregulating inhibitory receptors and increasing cytokine production [51]. The presence of T cell inhibitory signaling has been implicated in assistance with disease progression in glioblastomas thus further studies are warranted to further delineate the influence of BTK/BMX inhibition on T-cell infiltration, microglial behaviors and cytokine production within immunocompetent models [52].

Additionally, because S635 glioma cells orthotopically injected in immunocompetent hosts resulted in approximately 20–30 + days of survival with ibrutinib alone vs combination therapy, we were unable to assess long-term effects of ibrutinib therapy on disease course. Using another rat glioma model with less invasive quality and pronged survival without therapy could provide additional studies that help to explore systemic and neurologic sequelae from repeat ibrutinib therapy additive and/or synergistic with varied cytotoxic chemotherapy agents. These studies would assist in understanding more about the long-term effects of transient BBB disruption from ibrutinib as related to survival outcomes and tumor biology.

Other than S635, there only exists 9L, C6 and F98 rat derived glioma cells. We chose not to use 9L as it has previously been shown to be more akin histologically to gliosarcoma. C6 and F98 rat glioma lines, while most closely resemble (histologically and molecularly) glioblastoma, its growth patterns evoke alloimmunity or weak immunogenicity, respectively [53–55]. As such, its use has been cautioned for use with any evaluations that could aim to evaluate immunotherapeutics or agents that may influence the immune microenvironment [54]. As such, we opted to use a model with no limitations for evaluation. While the use of rodent models are highly necessary to advance to the neurooncologic field, there still exist restrictions within the suitability of models for translational applications.

Shi et al*.* reported that high BMX expression in glioma stem cells could be inhibited by ibrutinib resulting in decreased cell proliferation alone or with etoposide chemotherapeutic agent [14]. Comparatively, in this study, we found that ibrutinib has no significant effect on S635 rat glioma cell viability, yet it influenced endothelial junction proteins, inhibited efflux of ABCB1 and impaired migration. Classically, it is a known challenge to extrapolate in vitro dosing to rodent models due to the lack of dynamic fluidity as seen in a living system, thus we relied on published data from varied endothelial and cancer lines for treatment of our endothelial and glioma cells [12, 56, 57]. For additional clinical relevance, we investigated the additive effect of ibrutinib with and without doxil or doxorubicin treatment. Doxorubicin is a well-known substrate of both ABCB1 and ABCG2 and an effective chemotherapy drug that has been shown to hinder cancer proliferation of multiple solid tumors [58, 59]. However, doxorubicin cannot penetrate through the BBB easily and even with the assistance of ibrutinib we found no enhancement in survival or tumor volume with combined therapy (Additional file 2: Fig. 2). Doxil is the pegylated liposomal form of doxorubicin which confers a higher likelihood to cross the BBB, therefore these findings further point to the need to strategically select agents that can be paired with ibrutinib which are ABCB1 or potentially ABCC1 substrates and can impair glioma viability, migration and growth [60]. We evaluated free doxorubicin released from doxil within plasma and brain approximately 2 h after administration and found higher concentrations post ibrutinib. Yet, additional evaluations from 6 to 24 h after administration may aid determing differences in the brain and brain tumor settings regarding drug clearance with ibrutinib [24].

In our current study, we have provided evidence that ibrutinib influences brain endothelial integrity, efflux transport, and tumor progression. Our animal studies provide additional data in support of ibrutinib use to provide sustainable effects on BBB/BTB permeability and glioma cell propagation. Current clinical trials are ongoing to explore the use of ibrutinib with chemoradiation against glioblastoma which will provide further data on the use of this agent for varied treatment types (NCT03535350 and NCT05106296). Continued and novel research is needed to investigate ibrutinib’s efficacy with tumor directed agents, changes to the immune microenvironment and prolonged use for malignant gliomas; all with the aim of increasing CNS drug entry and improving disease survival.

## Supplementary Information

**Additional file 1.** High BTK expression seen in lymph node TMA tissue (positive control) compared with kidney, liver, colon, lung and soft tissue BTK staining (a). Decreased phospho-ERK expression seen after ibrutinib treatment of rat brain endothelium from 0.5-8 hours after treatment, **p<001 (b). Decreased ZO-1 tight junction expression on human brain endothelial cells seen with higher ibrutinib dosing (c). Ibrutinib significantly decreased rat brain endothelial cell-cell interaction after 4 hours treatment via electron microscopy imaging. 1. Mitochondria, 2. lysosome, 3. cell-cell junction, 4. herolysosome, 5. ribosome, 6. lamellar bodies (d). HEK overexpressing ABCB1/PGP transporter exhibited ibrutinib dose-dependently increased rhodamine accumulation, similar to valspodar (e).

**Additional file 2.** Treatment schema for animal model with ibrutinib and doxorubicin therapy (a). No statistical difference seen in model survival with doxorubicin alone or combination therapy (b). Alternative treatment schedules also provided no statistical difference in survival benefit with combination therapy, yet doxil alone provided a trend towards improved survival (c). Magnetic resonance imaging of control (left panel), doxil (left middle panel), ibrutinib (right middle panel) and combination (right panel) demonstrated no statistically significant difference in tumor volumes (d).

**Additional file 3.** qPCR Primers.

**Additional file 4.** Antibodies for Western blots and IF.

**Additional file 5.** Description of S635 glioma cell whole genome sequencing. Variant calls are described for select genes. For H3f3a, H3f3b, Idh1, Idh2, and Tert genes, variants called included only those that render a coding change but no amino acid change. Given this, the S635 glioma cell line can be characterized as IDH wild type. For Atrx, Egfr, and Tp53 genes, variants called included those that render both a coding change and a non-synonymous amino acid change. For Atrx, four single nucleotide variants were called (X:c.70901795:G>T:5.71%; X:c.70930985:G>T,7.41%; X:c.70931078:T>C,20.83%; X:c.70931082:G>C,10.53%) resulting in a non-synonymous impact for multiple transcripts (ENSRNOP00000070457:p.Ser1573Ile, ENSRNOP00000087612:p.Ser1584Ile, ENSRNOP00000087702:p.Ser1546Ile; ENSRNOP00000070457:p.Gly873Cys, ENSRNOP00000087612:p.Gly884Cys, ENSRNOP00000087702:p.Gly846Cys; ENSRNOP00000070457:p.Ser842Pro, ENSRNOP00000087612:p.Ser853Pro, ENSRNOP00000087702:p.Ser815Pro; ENSRNOP00000070457:p.Arg840Ser, ENSRNOP00000087612:p.Arg851Ser, ENSRNOP00000087702:p.Arg813Ser). For Egfr, two single nucleotide variants were called (14:c.91288218:A>G:15.38%; c.14:91341469:A>C) resulting in a non-synonymous impact for multiple transcripts (ENSRNOP00000006087:p.Arg132Gly, ENSRNOP00000078445:p.Arg106Gly, ENSRNOP00000080460:p.Arg106Gly; ENSRNOP00000080460:p.Lys940Thr) along with one insertion (14:91287423^91287424:->T:100%) that possibly impacts splicing for multiple transcripts (ENSRNOT00000006087:c.234+1dupT, ENSRNOT00000097681:c.159+28dupT, ENSRNOT00000111139:c.159+28dupT). For Tp53, two single nucleotide variants were called (10:c.54309391:C>A:6.90%, 10:c.54309411:C>A:8%) resulting in a non-synonymous impact for multiple transcripts (ENSRNOP00000047840:p.Ser313Tyr, ENSRNOP00000074031:p.Ser307Tyr, ENSRNOP00000075724:p.Ser313Tyr, ENSRNOP00000080907:p.Ser286Tyr, ENSRNOP00000089020:p.Ser321Tyr, ENSRNOP00000092831:p.Ser328Tyr; ENSRNOP00000047840:p.Pro320Thr, ENSRNOP00000074031:p.Pro314Thr, ENSRNOP00000075724:p.Pro320Thr, ENSRNOP00000080907:p.Pro293Thr, ENSRNOP00000089020:p.Pro328Thr, ENSRNOP00000092831:p.Pro335Thr).

**Additional file 6.** S635 glioma cell whole genome sequencing table.

## References

[1] Arvanitis CD, Ferraro GB, Jain RK (2020) The blood-brain barrier and blood-tumour barrier in brain tumours and metastases. Nat Rev Cancer 20(1):26–41 31601988 10.1038/s41568-019-0205-x PMC8246629

[2] Belykh E, Shaffer KV, Lin C, Byvaltsev VA, Preul MC, Chen L (2020) Blood-brain barrier, blood-brain tumor barrier, and fluorescence-guided neurosurgical oncology: delivering optical labels to brain tumors. Front Oncol 10:739 32582530 10.3389/fonc.2020.00739 PMC7290051

[3] van Tellingen O, Yetkin-Arik B, de Gooijer MC, Wesseling P, Wurdinger T, de Vries HE (2015) Overcoming the blood-brain tumor barrier for effective glioblastoma treatment. Drug Resist Updates Rev Comment Antimicrob Anticancer Chemotherapy 19:1–12 10.1016/j.drup.2015.02.002 25791797

[4] Abbott NJ, Patabendige AA, Dolman DE, Yusof SR, Begley DJ (2010) Structure and function of the blood-brain barrier. Neurobiol Dis 37(1):13–25 19664713 10.1016/j.nbd.2009.07.030

[5] Daneman R, Agalliu D, Zhou L, Kuhnert F, Kuo CJ, Barres BA (2009) Wnt/beta-catenin signaling is required for CNS, but not non-CNS, angiogenesis. Proc Natl Acad Sci U S A 106(2):641–646 19129494 10.1073/pnas.0805165106 PMC2626756

[6] Saunders NR, Habgood MD, Mollgard K, Dziegielewska KM (2016) The biological significance of brain barrier mechanisms: Help or hindrance in drug delivery to the central nervous system? F1000Res 5:313 10.12688/f1000research.7378.1 PMC4786902 26998242

[7] Shen S, Zhang W (2010) ABC transporters and drug efflux at the blood-brain barrier. Rev Neurosci 21(1):29–53 20458886 10.1515/revneuro.2010.21.1.29

[8] Zhang W, Mojsilovic-Petrovic J, Andrade MF, Zhang H, Ball M, Stanimirovic DB (2003) The expression and functional characterization of ABCG2 in brain endothelial cells and vessels. FASEB J 17(14):2085–2087 12958161 10.1096/fj.02-1131fje

[9] de Gooijer MC, Kemper EM, Buil LCM, Citirikkaya CH, Buckle T, Beijnen JH, van Tellingen O (2021) ATP-binding cassette transporters restrict drug delivery and efficacy against brain tumors even when blood-brain barrier integrity is lost. Cell Rep Med 2(1):100184 33521698 10.1016/j.xcrm.2020.100184 PMC7817868

[10] Banks WA (2009) Characteristics of compounds that cross the blood-brain barrier. BMC Neurol 9(Suppl 1):S3 19534732 10.1186/1471-2377-9-S1-S3 PMC2697631

[11] Gu JJ, Zhang JH, Chen HJ, Wang SS (2016) TPX2 promotes glioma cell proliferation and invasion via activation of the AKT signaling pathway. Oncol Lett 12(6):5015–5022 28105208 10.3892/ol.2016.5371 PMC5228448

[12] Kohs TCL, Olson SR, Pang J, Jordan KR, Zheng TJ, Xie A, Hodovan J, Muller M, McArthur C, Johnson J, Sousa BB, Wallisch M, Kievit P, Aslan JE, Seixas JD, Bernardes GJL, Hinds MT, Lindner JR, McCarty OJT, Puy C, Shatzel JJ (2022) Ibrutinib inhibits BMX-dependent endothelial VCAM-1 expression in vitro and pro-atherosclerotic endothelial activation and platelet adhesion in vivo. Cell Mol Bioeng 15(3):231–243 35611166 10.1007/s12195-022-00723-1 PMC9124262

[13] Grommes C, Pastore A, Palaskas N, Tang SS, Campos C, Schartz D, Codega P, Nichol D, Clark O, Hsieh WY, Rohle D, Rosenblum M, Viale A, Tabar VS, Brennan CW, Gavrilovic IT, Kaley TJ, Nolan CP, Omuro A, Pentsova E, Thomas AA, Tsyvkin E, Noy A, Palomba ML, Hamlin P, Sauter CS, Moskowitz CH, Wolfe J, Dogan A, Won M, Glass J, Peak S, Lallana EC, Hatzoglou V, Reiner AS, Gutin PH, Huse JT, Panageas KS, Graeber TG, Schultz N, DeAngelis LM, Mellinghoff IK (2017) Ibrutinib unmasks critical role of bruton tyrosine kinase in primary CNS lymphoma. Cancer Discov 7(9):1018–1029 28619981 10.1158/2159-8290.CD-17-0613 PMC5581705

[14] Shi Y, Guryanova OA, Zhou W, Liu C, Huang Z, Fang X, Wang X, Chen C, Wu Q, He Z, Wang W, Zhang W, Jiang T, Liu Q, Chen Y, Wang W, Wu J, Kim L, Gimple RC, Feng H, Kung HF, Yu JS, Rich JN, Ping YF, Bian XW, Bao S (2018) Ibrutinib inactivates BMX-STAT3 in glioma stem cells to impair malignant growth and radioresistance. Sci Transl Med 10(443):eaah6816 29848664 10.1126/scitranslmed.aah6816 PMC6431250

[15] Guerra DAP, Paiva AE, Sena IFG, Azevedo PO, Silva WN, Mintz A, Birbrair A (2018) Targeting glioblastoma-derived pericytes improves chemotherapeutic outcome. Angiogenesis 21(4):667–675 29761249 10.1007/s10456-018-9621-x PMC6238207

[16] Zhou W, Chen C, Shi Y, Wu Q, Gimple RC, Fang X, Huang Z, Zhai K, Ke SQ, Ping YF, Feng H, Rich JN, Yu JS, Bao S, Bian XW (2017) Targeting glioma stem cell-derived pericytes disrupts the blood-tumor barrier and improves chemotherapeutic efficacy. Cell Stem Cell 21(5):591–603 29100012 10.1016/j.stem.2017.10.002 PMC5687837

[17] Zhang H, Patel A, Wang YJ, Zhang YK, Kathawala RJ, Qiu LH, Patel BA, Huang LH, Shukla S, Yang DH, Ambudkar SV, Fu LW, Chen ZS (2017) The BTK inhibitor ibrutinib (PCI-32765) overcomes paclitaxel resistance in ABCB1- and ABCC10-overexpressing cells and tumors. Mol Cancer Ther 16(6):1021–1030 28265007 10.1158/1535-7163.MCT-16-0511 PMC7830771

[18] Lee YS, Bigner SH, Eng LF, Molnar P, Kuruvilla A, Groothuis DR, Bigner DD (1986) A glial fibrillary acidic protein-expressing and tumorigenic cell line derived from an avian sarcoma virus-induced rat astrocytoma. J Neuropathol Exp Neurol 45(6):704–720 3021915 10.1097/00005072-198611000-00008

[19] Robey RW, Shukla S, Finley EM, Oldham RK, Barnett D, Ambudkar SV, Fojo T, Bates SE (2008) Inhibition of P-glycoprotein (ABCB1)- and multidrug resistance-associated protein 1 (ABCC1)-mediated transport by the orally administered inhibitor, CBT-1((R)). Biochem Pharmacol 75(6):1302–1312 18234154 10.1016/j.bcp.2007.12.001 PMC2346578

[20] Vézina A, Manglani M, Morris D, Foster B, McCord M, Song H, Zhang M, Davis D, Zhang W, Bills J, Nagashima K, Shankarappa P, Kindrick J, Walbridge S, Peer CJ, Figg WD, Gilbert MR, McGavern DB, Muldoon LL, Jackson S (2021) Adenosine A2A receptor activation enhances blood-tumor barrier permeability in a rodent glioma model. Mol Cancer Res MCR 19(12):2081–2095 34521765 10.1158/1541-7786.MCR-19-0995 PMC8642293

[21] Nie W, Zan X, Yu T, Ran M, Hong Z, He Y, Yang T, Ju Y, Gao X (2020) Synergetic therapy of glioma mediated by a dual delivery system loading α-mangostin and doxorubicin through cell cycle arrest and apoptotic pathways. Cell Death Dis 11(10):928 33116114 10.1038/s41419-020-03133-1 PMC7595144

[22] Watanabe A, Murayama S, Karasawa K, Yamamoto E, Morikawa S, Takita R, Murata S, Kato M (2019) A simple and easy method of monitoring doxorubicin release from a liposomal drug formulation in the serum using fluorescence spectroscopy. Chem Pharm Bull 67(4):367–371 10.1248/cpb.c18-00868 30930441

[23] Itoh N, Kimoto A, Yamamoto E, Higashi T, Santa T, Funatsu T, Kato M (2017) High performance liquid chromatography analysis of 100-nm liposomal nanoparticles using polymer-coated, silica monolithic columns with aqueous mobile phase. J Chromatogr A 1484:34–40 28089273 10.1016/j.chroma.2016.12.080

[24] Yamamoto E, Hyodo K, Suzuki T, Ishihara H, Kikuchi H, Kato M (2018) Simulation of stimuli-responsive and stoichiometrically controlled release rate of doxorubicin from liposomes in tumor interstitial fluid. Pharm Res 35(5):103 29557075 10.1007/s11095-018-2380-y

[25] Mauda-Havakuk M, Mikhail AS, Starost MF, Jones EC, Karim B, Kleiner DE, Partanen A, Esparza-Trujillo JA, Bakhutashvili I, Wakim PG, Kassin MT, Lewis AL, Karanian JW, Wood BJ, Pritchard WF (2021) Imaging pathology, and immune correlates in the woodchuck hepatic tumor model. J Hepatocell Carcinoma 8:71–83 33728278 10.2147/JHC.S287800 PMC7955744

[26] Ames HM, Rooper LM, Laterra JJ, Eberhart CG, Rodriguez FJ (2018) INSM1 expression is frequent in primary central nervous system neoplasms but not in the adult brain parenchyma. J Neuropathol Exp Neurol 77(5):374–382 29490065 10.1093/jnen/nly014 PMC6019041

[27] Fedor HL, De Marzo AM (2005) Practical methods for tissue microarray construction. Methods Mol Med 103:89–101 15542899 10.1385/1-59259-780-7:089

[28] Wei L, Su YK, Lin CM, Chao TY, Huang SP, Huynh TT, Jan HJ, Whang-Peng J, Chiou JF, Wu AT, Hsiao M (2016) Preclinical investigation of ibrutinib, a Bruton’s kinase tyrosine (Btk) inhibitor, in suppressing glioma tumorigenesis and stem cell phenotypes. Oncotarget 7(43):69961–69975 27564106 10.18632/oncotarget.11572 PMC5342527

[29] Yue C, Niu M, Shan QQ, Zhou T, Tu Y, Xie P, Hua L, Yu R, Liu X (2017) High expression of Bruton’s tyrosine kinase (BTK) is required for EGFR-induced NF-kappaB activation and predicts poor prognosis in human glioma. J Exp Clin Cancer Res CR 36(1):132 28946903 10.1186/s13046-017-0600-7 PMC5613332

[30] Zhao Z, Meng F, Wang W, Wang Z, Zhang C, Jiang T (2017) Comprehensive RNA-seq transcriptomic profiling in the malignant progression of gliomas. Sci Data 4:170024 28291232 10.1038/sdata.2017.24 PMC5349247

[31] Zhang Q, Zheng M, Betancourt CE, Liu L, Sitikov A, Sladojevic N, Zhao Q, Zhang JH, Liao JK, Wu R (2021) Increase in blood-brain barrier (BBB) permeability is regulated by MMP3 via the ERK signaling pathway. Oxid Med Cell Longev 2021:6655122 33859779 10.1155/2021/6655122 PMC8026308

[32] Ryu WI, Lee H, Bae HC, Jeon J, Ryu HJ, Kim J, Kim JH, Son JW, Kim J, Imai Y, Yamanishi K, Jeong SH, Son SW (2018) IL-33 down-regulates CLDN1 expression through the ERK/STAT3 pathway in keratinocytes. J Dermatol Sci 90(3):313–322 29534857 10.1016/j.jdermsci.2018.02.017

[33] Chu H, Yang X, Huang C, Gao Z, Tang Y, Dong Q (2017) Apelin-13 protects against ischemic blood-brain barrier damage through the effects of aquaporin-4. Cerebrovasc Dis 44(1–2):10–25 28402976 10.1159/000460261

[34] Shi Y, Guryanova OA, Zhou W, Liu C, Huang Z, Fang X, Wang X, Chen C, Wu Q, He Z, Wang W, Zhang W, Jiang T, Liu Q, Chen Y, Wang W, Wu J, Kim L, Gimple RC, Feng H, Kung HF, Yu JS, Rich JN, Ping YF, Bian XW, Bao S (2018) Ibrutinib inactivates BMX-STAT3 in glioma stem cells to impair malignant growth and radioresistance. Sci Transl Med 10(443):eaah6816 29848664 10.1126/scitranslmed.aah6816 PMC6431250

[35] Hatoum A, Mohammed R, Zakieh O (2019) The unique invasiveness of glioblastoma and possible drug targets on extracellular matrix. Cancer Manag Res 11:1843–1855 30881112 10.2147/CMAR.S186142 PMC6395056

[36] Davis ME (2016) Glioblastoma: overview of disease and treatment. Clin J Oncol Nurs 20(5 Suppl):S2–S8 27668386 10.1188/16.CJON.S1.2-8 PMC5123811

[37] Fernandes C, Costa A, Osorio L, Lago RC, Linhares P, Carvalho B, Caeiro C (2017) Current standards of care in glioblastoma therapy. In: De Vleeschouwer S (ed) Glioblastoma. Codon Publications, Brisbane (AU) 29251860

[38] Minniti G, Niyazi M, Alongi F, Navarria P, Belka C (2021) Current status and recent advances in reirradiation of glioblastoma. Radiat Oncol 16(1):36 33602305 10.1186/s13014-021-01767-9 PMC7890828

[39] Wang Z, Sun H, Yakisich JS (2014) Overcoming the blood-brain barrier for chemotherapy: limitations, challenges and rising problems. Anticancer Agents Med Chem 14(8):1085–1093 23092271 10.2174/18715206113139990029

[40] Portnow J, Badie B, Chen M, Liu A, Blanchard S, Synold TW (2009) The neuropharmacokinetics of temozolomide in patients with resectable brain tumors: potential implications for the current approach to chemoradiation. Clin Cancer Res 15(22):7092–7098 19861433 10.1158/1078-0432.CCR-09-1349 PMC2908372

[41] Jackson S, Weingart J, Nduom EK, Harfi TT, George RT, McAreavey D, Ye X, Anders NM, Peer C, Figg WD, Gilbert M, Rudek MA, Grossman SA (2018) The effect of an adenosine A(2A) agonist on intra-tumoral concentrations of temozolomide in patients with recurrent glioblastoma. Fluids Barriers CNS 15(1):2 29332604 10.1186/s12987-017-0088-8 PMC5767971

[42] Proescholdt MA, Merrill MJ, Ikejiri B, Walbridge S, Akbasak A, Jacobson S, Oldfield EH (2001) Site-specific immune response to implanted gliomas. J Neurosurg 95(6):1012–1019 11765816 10.3171/jns.2001.95.6.1012

[43] Falter J, Lohmeier A, Eberl P, Stoerr EM, Koskimäki J, Falter L, Rossmann J, Mederer T, Schmidt NO, Proescholdt M (2023) CXCR2-blocking has context-sensitive effects on rat glioblastoma cell line outgrowth (S635) in an organotypic rat brain slice culture depending on microglia-depletion (PLX5622) and dexamethasone treatment. Int J Mol Sci 24(23):16803 38069130 10.3390/ijms242316803 PMC10706712

[44] Fu Y, Huang R, Zheng Y, Zhang Z, Liang A (2011) Glioma-derived mutations in isocitrate dehydrogenase 2 beneficial to traditional chemotherapy. Biochem Biophys Res Commun 410(2):218–223 21641335 10.1016/j.bbrc.2011.05.108

[45] He Y, Luo Y, Tang S, Rajantie I, Salven P, Heil M, Zhang R, Luo D, Li X, Chi H, Yu J, Carmeliet P, Schaper W, Sinusas AJ, Sessa WC, Alitalo K, Min W (2006) Critical function of Bmx/Etk in ischemia-mediated arteriogenesis and angiogenesis. J Clin Investig 116(9):2344–2355 16932810 10.1172/JCI28123 PMC1551932

[46] Holopainen T, Rasanen M, Anisimov A, Tuomainen T, Zheng W, Tvorogov D, Hulmi JJ, Andersson LC, Cenni B, Tavi P, Mervaala E, Kivela R, Alitalo K (2015) Endothelial Bmx tyrosine kinase activity is essential for myocardial hypertrophy and remodeling. Proc Natl Acad Sci U S A 112(42):13063–13068 26430242 10.1073/pnas.1517810112 PMC4620883

[47] Dai B, Kim O, Xie Y, Guo Z, Xu K, Wang B, Kong X, Melamed J, Chen H, Bieberich CJ, Borowsky AD, Kung HJ, Wei G, Ostrowski MC, Brodie A, Qiu Y (2006) Tyrosine kinase Etk/BMX is up-regulated in human prostate cancer and its overexpression induces prostate intraepithelial neoplasia in mouse. Cancer Res 66(16):8058–8064 16912182 10.1158/0008-5472.CAN-06-1364

[48] Abassi YA, Rehn M, Ekman N, Alitalo K, Vuori K (2003) p130Cas Couples the tyrosine kinase Bmx/Etk with regulation of the actin cytoskeleton and cell migration. J Biol Chem 278(37):35636–35643 12832404 10.1074/jbc.M306438200

[49] Chau CH, Clavijo CA, Deng HT, Zhang Q, Kim KJ, Qiu Y, Le AD, Ann DK (2005) Etk/Bmx mediates expression of stress-induced adaptive genes VEGF, PAI-1, and iNOS via multiple signaling cascades in different cell systems. Am J Physiol Cell Physiol 289(2):C444–C454 15788485 10.1152/ajpcell.00410.2004

[50] Kim B, Breton S (2016) The MAPK/ERK-signaling pathway regulates the expression and distribution of tight junction proteins in the mouse proximal epididymis. Biol Reprod 94(1):22 26658708 10.1095/biolreprod.115.134965 PMC4809559

[51] Li L, Zhao M, Kiernan CH, Castro Eiro MD, van Meurs M, Brouwers-Haspels I, Wilmsen MEP, Grashof DGB, van de Werken HJG, Hendriks RW, Mueller YM, Katsikis PD (2023) Ibrutinib directly reduces CD8+T cell exhaustion independent of BTK. Front Immunol 14:1201415 37771591 10.3389/fimmu.2023.1201415 PMC10523025

[52] Lee J, Nicosia M, Hong ES, Silver DJ, Li C, Bayik D, Watson DC, Lauko A, Kay KE, Wang SZ, Johnson S, McGraw M, Grabowski MM, Kish DD, Desai AB, Goodman WA, Cameron SJ, Okada H, Valujskikh A, Fairchild RL, Ahluwalia MS, Lathia JD (2023) Sex-biased T-cell exhaustion drives differential immune responses in glioblastoma. Cancer Discov 13(9):2090–2105 37378557 10.1158/2159-8290.CD-22-0869 PMC10481130

[53] Giakoumettis D, Kritis A, Foroglou N (2018) C6 cell line: the gold standard in glioma research. Hippokratia 22(3):105–112 31641331 PMC6801124

[54] Parsa AT, Chakrabarti I, Hurley PT, Chi JH, Hall JS, Kaiser MG, Bruce JN (2000) Limitations of the C6/Wistar rat intracerebral glioma model: implications for evaluating immunotherapy. Neurosurgery 47(4):993–9 11014444 10.1097/00006123-200010000-00050

[55] Sahu U, Barth RF, Otani Y, McCormack R, Kaur B (2022) Rat and mouse brain tumor models for experimental neuro-oncology research. J Neuropathol Exp Neurol 81(5):312–329 35446393 10.1093/jnen/nlac021 PMC9113334

[56] Liu J, Liu Z, Zhang J, Chen X, Chen J, Sui L, Yu J (2022) Ibrutinib inhibits angiogenesis and tumorigenesis in a BTK-independent manner. Pharmaceutics 14(9):1876 36145624 10.3390/pharmaceutics14091876 PMC9506105

[57] Segura-Collar B, Garranzo-Asensio M, Herranz B, Hernández-SanMiguel E, Cejalvo T, Casas BS, Matheu A, Pérez-Núñez Á, Sepúlveda-Sánchez JM, Hernández-Laín A, Palma V, Gargini R, Sánchez-Gómez P (2021) Tumor-derived pericytes driven by EGFR mutations govern the vascular and immune microenvironment of gliomas. Cancer Res 81(8):2142–2156 33593822 10.1158/0008-5472.CAN-20-3558

[58] Meredith AM, Dass CR (2016) Increasing role of the cancer chemotherapeutic doxorubicin in cellular metabolism. J Pharm Pharmacol 68(6):729–741 26989862 10.1111/jphp.12539

[59] Rivankar S (2014) An overview of doxorubicin formulations in cancer therapy. J Cancer Res Ther 10(4):853–858 25579518 10.4103/0973-1482.139267

[60] Gaillard PJ, Appeldoorn CC, Dorland R, van Kregten J, Manca F, Vugts DJ, Windhorst B, van Dongen GA, de Vries HE, Maussang D, van Tellingen O (2014) Pharmacokinetics, brain delivery, and efficacy in brain tumor-bearing mice of glutathione pegylated liposomal doxorubicin (2B3-101). PLoS ONE 9(1):e82331 24416140 10.1371/journal.pone.0082331 PMC3885379

