# Supplementary material for: Ibrutinib disrupts blood-tumor barrier integrity and prolongs survival in rodent glioma model
Source: Acta Neuropathol Commun. 2024 Apr 8;12:56. doi: 10.1186/s40478-024-01763-6 (PMC11003129; doi:10.1186/s40478-024-01763-6)
Supplement: Supplementary file 3 — Additional file 3. qPCR Primers. [file 40478_2024_1763_MOESM3_ESM.docx]

**Supplemental Table 1.** qPCR Primers

| **Gene** | **Company** | **Assay ID** |
| --- | --- | --- |
| LsR | Bio-Rad | qRnoCED0020018 |
| Ocel1 | Bio-Rad | qRnoCED0011780 |
| Cldn1 | Bio-Rad | qRnoCED0051349 |
| Cldn3 | Bio-Rad | qRnoCED0052933 |
| Cldn5 | Bio-Rad | qRnoCED000686 |
| Tjp1 | Bio-Rad | qRnoCID0001801 |
| Marveld2 | Bio-Rad | qRnoCED0018999 |
| S18 | Bio-Rad | qRnoCED0003920 |
| BTK | Thermo Fisher | Rn01472231_m1 |
| S18 | Thermo Fisher | Rn01428913_gh |
