# Supplementary material for: Ibrutinib disrupts blood-tumor barrier integrity and prolongs survival in rodent glioma model
Source: Acta Neuropathol Commun. 2024 Apr 8;12:56. doi: 10.1186/s40478-024-01763-6 (PMC11003129; doi:10.1186/s40478-024-01763-6)
Supplement: Supplementary file 4 — Additional file 4. Antibodies for Western blots and IF. [file 40478_2024_1763_MOESM4_ESM.dotx]

**Supplemental Table 2.** Antibodies for Western blots and IF

| **Antibody** | **Company** | **Catalog #** | **WB Dilution** | **Primary** |
| --- | --- | --- | --- | --- |
| GAPDH | Cell signaling | 97116 | 1:10000 | Mouse |
| ZO-1 | Thermo Fisher | 40-2200 | 1:1000 | Rabbit |
| ZO-1 | Thermo Fisher | 33-9100 | 1:200 | Rabbit |
| Claudin3 | Thermo Fisher | PAS-16887 | 1:1000 | Rabbit |
| Marveld2/Tricellulin | Thermo Fisher | 48-8400 | 1:1000 | Rabbit |
| CD31/PECAM | Abcam | ab28364 | 1:100 | Rabbit |
| Claudin5 | Invitrogen | 35-2500 | 1:50 | Mouse |
| IRDye 6800RD  Goat anti-Mouse IGg | Li-Cor | 926-68070 | 1:10000 | Mouse |
| IRDye800CW  Donkey anti-Rabbit | Li-Cor | 926-32211 | 1:10000 | Rabbit |
| Alexa Fluor 594 goat anti-mouse | Invitrogen | A11005 | 1:200 | Mouse |
