# Supplementary material for: Ibrutinib disrupts blood-tumor barrier integrity and prolongs survival in rodent glioma model
Source: Acta Neuropathol Commun. 2024 Apr 8;12:56. doi: 10.1186/s40478-024-01763-6 (PMC11003129; doi:10.1186/s40478-024-01763-6)
Supplement: Supplementary file 5 — Additional file 5. Description of S635 glioma cell whole genome sequencing. Variant calls are described for select genes. For H3f3a, H3f3b, Idh1, Idh2, and Tert genes, variants called included only those that render a coding change but no amino acid change. Given this, the S635 glioma cell line can be characterized as IDH wild type. For Atrx, Egfr, and Tp53 genes, variants called included those that render both a coding change and a non-synonymous amino acid change. For Atrx, four single nucleotide variants were called (X:c.70901795:G>T:5.71%; X:c.70930985:G>T,7.41%; X:c.70931078:T>C,20.83%; X:c.70931082:G>C,10.53%) resulting in a non-synonymous impact for multiple transcripts (ENSRNOP00000070457:p.Ser1573Ile, ENSRNOP00000087612:p.Ser1584Ile, ENSRNOP00000087702:p.Ser1546Ile; ENSRNOP00000070457:p.Gly873Cys, ENSRNOP00000087612:p.Gly884Cys, ENSRNOP00000087702:p.Gly846Cys; ENSRNOP00000070457:p.Ser842Pro, ENSRNOP00000087612:p.Ser853Pro, ENSRNOP00000087702:p.Ser815Pro; ENSRNOP00000070457:p.Arg840Ser, ENSRNOP00000087612:p.Arg851Ser, ENSRNOP00000087702:p.Arg813Ser). For Egfr, two single nucleotide variants were called (14:c.91288218:A>G:15.38%; c.14:91341469:A>C) resulting in a non-synonymous impact for multiple transcripts (ENSRNOP00000006087:p.Arg132Gly, ENSRNOP00000078445:p.Arg106Gly, ENSRNOP00000080460:p.Arg106Gly; ENSRNOP00000080460:p.Lys940Thr) along with one insertion (14:91287423^91287424:->T:100%) that possibly impacts splicing for multiple transcripts (ENSRNOT00000006087:c.234+1dupT, ENSRNOT00000097681:c.159+28dupT, ENSRNOT00000111139:c.159+28dupT). For Tp53, two single nucleotide variants were called (10:c.54309391:C>A:6.90%, 10:c.54309411:C>A:8%) resulting in a non-synonymous impact for multiple transcripts (ENSRNOP00000047840:p.Ser313Tyr, ENSRNOP00000074031:p.Ser307Tyr, ENSRNOP00000075724:p.Ser313Tyr, ENSRNOP00000080907:p.Ser286Tyr, ENSRNOP00000089020:p.Ser321Tyr, ENSRNOP00000092831:p.Ser328Tyr; ENSRNOP00000047840:p.Pro320Thr, ENSRNOP00000074031:p.Pro314Thr, EN [file 40478_2024_1763_MOESM5_ESM.docx]

**Supplementary Table 3**

Variant calls are described for select genes. For H3f3a, H3f3b, Idh1, Idh2, and Tert genes, variants called included only those that render a coding change but no amino acid change. Given this, the S635 glioma cell line can be characterized as IDH wild type. For Atrx, Egfr, and Tp53 genes, variants called included those that render both a coding change and a non-synonymous amino acid change. For Atrx, four single nucleotide variants were called (X:c.70901795:G>T:5.71%; X:c.70930985:G>T,7.41%; X:c.70931078:T>C,20.83%; X:c.70931082:G>C,10.53%) resulting in a non-synonymous impact for multiple transcripts (ENSRNOP00000070457:p.Ser1573Ile, ENSRNOP00000087612:p.Ser1584Ile, ENSRNOP00000087702:p.Ser1546Ile; ENSRNOP00000070457:p.Gly873Cys, ENSRNOP00000087612:p.Gly884Cys, ENSRNOP00000087702:p.Gly846Cys; ENSRNOP00000070457:p.Ser842Pro, ENSRNOP00000087612:p.Ser853Pro, ENSRNOP00000087702:p.Ser815Pro; ENSRNOP00000070457:p.Arg840Ser, ENSRNOP00000087612:p.Arg851Ser, ENSRNOP00000087702:p.Arg813Ser). For Egfr, two single nucleotide variants were called (14:c.91288218:A>G:15.38%; c.14:91341469:A>C) resulting in a non-synonymous impact for multiple transcripts (ENSRNOP00000006087:p.Arg132Gly, ENSRNOP00000078445:p.Arg106Gly, ENSRNOP00000080460:p.Arg106Gly; ENSRNOP00000080460:p.Lys940Thr) along with one insertion (14:91287423^91287424:->T:100%) that possibly impacts splicing for multiple transcripts (ENSRNOT00000006087:c.234+1dupT, ENSRNOT00000097681:c.159+28dupT, ENSRNOT00000111139:c.159+28dupT). For Tp53, two single nucleotide variants were called (10:c.54309391:C>A:6.90%, 10:c.54309411:C>A:8%) resulting in a non-synonymous impact for multiple transcripts (ENSRNOP00000047840:p.Ser313Tyr, ENSRNOP00000074031:p.Ser307Tyr, ENSRNOP00000075724:p.Ser313Tyr, ENSRNOP00000080907:p.Ser286Tyr, ENSRNOP00000089020:p.Ser321Tyr, ENSRNOP00000092831:p.Ser328Tyr; ENSRNOP00000047840:p.Pro320Thr, ENSRNOP00000074031:p.Pro314Thr, ENSRNOP00000075724:p.Pro320Thr, ENSRNOP00000080907:p.Pro293Thr, ENSRNOP00000089020:p.Pro328Thr, ENSRNOP00000092831:p.Pro335Thr).
